# A Cyclic Peptidic Serine Protease Inhibitor: Increasing Affinity by Increasing Peptide Flexibility

**DOI:** 10.1371/journal.pone.0115872

**Published:** 2014-12-29

**Authors:** Baoyu Zhao, Peng Xu, Longguang Jiang, Berit Paaske, Tobias Kromann-Hansen, Jan K. Jensen, Hans Peter Sørensen, Zhuo Liu, Jakob T. Nielsen, Anni Christensen, Masood Hosseini, Kasper K. Sørensen, Niels Christian Nielsen, Knud J. Jensen, Mingdong Huang, Peter A. Andreasen

**Affiliations:** 1 Danish-Chinese Centre for Proteases and Cancer, Fujian Institute of Research on the Structure of Matter, Chinese Academy of Sciences, Fuzhou, China; 2 Danish-Chinese Centre for Proteases and Cancer, Department of Molecular Biology and Genetics, Aarhus University, Aarhus, Denmark; 3 Nanoscience Center and Department of Chemistry, University of Aarhus, Aarhus, Denmark; 4 Department of Chemistry, Faculty of Science, University of Copenhagen, Copenhagen, Denmark; University of Helsinki, Finland

## Abstract

Peptides are attracting increasing interest as protease inhibitors. Here, we demonstrate a new inhibitory mechanism and a new type of exosite interactions for a phage-displayed peptide library-derived competitive inhibitor, mupain-1 (CPAYSRYLDC), of the serine protease murine urokinase-type plasminogen activator (uPA). We used X-ray crystal structure analysis, site-directed mutagenesis, liquid state NMR, surface plasmon resonance analysis, and isothermal titration calorimetry and wild type and engineered variants of murine and human uPA. We demonstrate that Arg^6^ inserts into the S1 specificity pocket, its carbonyl group aligning improperly relative to Ser^195^ and the oxyanion hole, explaining why the peptide is an inhibitor rather than a substrate. Substitution of the P1 Arg with novel unnatural Arg analogues with aliphatic or aromatic ring structures led to an increased affinity, depending on changes in both P1 - S1 and exosite interactions. Site-directed mutagenesis showed that exosite interactions, while still supporting high affinity binding, differed substantially between different uPA variants. Surprisingly, high affinity binding was facilitated by Ala-substitution of Asp^9^ of the peptide, in spite of a less favorable binding entropy and loss of a polar interaction. We conclude that increased flexibility of the peptide allows more favorable exosite interactions, which, in combination with the use of novel Arg analogues as P1 residues, can be used to manipulate the affinity and specificity of this peptidic inhibitor, a concept different from conventional attempts at improving inhibitor affinity by reducing the entropic burden.

## Introduction

Peptides are of considerable interest as drug candidates. Peptides binding to specific protein targets can be selected from phage-displayed peptide libraries with a diversity of up to 10^6^ different sequences. The primary structure of the peptides in the libraries can be modified by introduction of disulfide bonds [1] or by chemical cross-linking [2]. Peptides directly selected from phage-displayed peptide libraries usually bind their targets with *K*_D_ values in the µM range, but the affinities can be improved by construction of focused libraries or chemical modification, like introduction of unnatural amino acids. Peptides have predictable absorption, distribution, metabolism, and excretion properties, can be delivered *in vivo* by new formulation methods, and stabilized against proteolytic degradation by various means [3].

Serine proteases of the trypsin family (clan SA) have many physiological and pathophysiological functions [4]–[6]. There is therefore extensive interest in generating specific inhibitors for pharmacological intervention with their enzymatic activity. Moreover, serine proteases are classical subjects for studies of catalytic and inhibitory mechanisms [7]. One interesting member of the trypsin family of serine proteases is urokinase-type plasminogen activator (uPA), which catalyses the conversion of the zymogen plasminogen into the active protease plasmin through cleavage of plasminogen's Arg^15^–Val^16^ bond (using the chymotrypsin numbering [8]). Plasmin generated by uPA participates in the turnover of extracellular matrix proteins in physiological and pathophysiological tissue remodeling [9], [10]. Abnormal expression of uPA is responsible for tissue damage in several pathological conditions, including rheumatoid arthritis, allergic vasculitis, and xeroderma pigmentosum, and in particular, is a key factor for the invasive capacity of malignant tumors [11]. uPA is therefore a potential therapeutic target.

**Figure 1:**
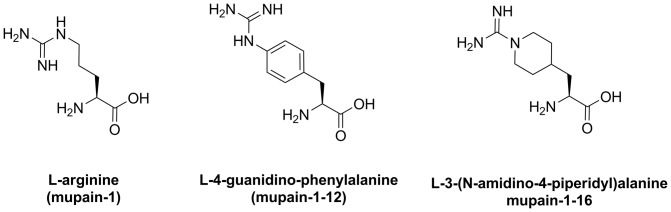
The structures of the P1 amino acids being studied here.

From a phage-displayed peptide library, we previously isolated a serum-stable, disulfide bond-constrained peptide, CPAYSRYLDC, termed mupain-1, which competitively inhibits murine uPA (muPA). As based on site-directed mutagenesis, mupain-1 gains high specificity for its target by having an extended interaction surface with the target protease, involving a number of exosite interactions. Its affinity for the target is moderate, the *K*_i_ value for inhibition of muPA being around 0.5 µM [12]. Substituting the P1 Arg residue with different non-natural amino acids in a mupain-1 background improved the affinity. Two variants of mupain-1, with the unnatural amino acids L-4-guanidino-phenylalanine or L-3-(N-amidino-4-piperidyl)alanine (Fig. 1) as P1 residues instead of the original Arg, have a 2- to 10-fold improved affinities [13].

In this study, we aimed at understanding the inhibitory mechanism and binding mechanism of mupain-1 and its derivatives. Why are these peptides protease inhibitors and not protease substrates? Which are the molecular events during the binding of peptides to serine proteases? Why do P1 substitutions increase the affinity? Is the specificity of the peptides among different serine proteases determined by the fit of the P1 residue into the specificity pocket, the exosite interactions, or the solution structures?

To answer these questions, we used X-ray crystal structure analysis, site-directed mutagenesis, surface plasmon resonance (SPR), isothermal titration calorimetry (ITC), and NMR spectroscopy to study the interaction of mupain-1 and derivatives with recombinant wild type (wt) muPA and engineered variants of muPA and human uPA (huPA). Several recent papers on peptidic protease inhibitors describe how binding affinity can be increased by a more favorable binding entropy following introduction of a more rigid peptide structure by bicyclisation [2], [14], [15]. Here, we go in another direction and show how increased flexibility can lead to an increased affinity.

## Materials and Methods

### Peptides

Chemicals for peptide synthesis were purchased from Sigma-Aldrich, Iris Biotech GmbH, or Rapp Polymere GmbH, and used without further purification. Fmoc-L-4-guanidino-phenylalanine(*N*, *N*′-di-Boc)-OH and Fmoc-L-Ala-4-piperidyl(Alloc)-OH were commercially available. Analytical HPLC was performed on a Dionex UltiMate 3000, using a Phenomenex Gemini 110 Å C18 column (3 µm, 4.6×50 mm) with a flow rate of 1.0 ml per min and a linear gradient going from 95% H_2_O, 5% acetonitrile with 0.1% HCOOH to 100% acetonitrile with 0.1% HCOOH over 10 min. Preparative HPLC was performed using a Dionex UltiMate 3000, equipped with a Phenomenex Gemini-NX C18 110 Å column, running at a flow rate of 10.0 ml/min and a linear gradient going from 95% H_2_O/5% acetonitrile with 0.1% TFA to 100% acetonitrile with 0.1% TFA over 30 min. High resolution mass spectra were obtained on a Micromass LCT high resolution time-of-flight instrument by direct injection. Ionization was performed in positive electrospray mode.

Solid-phase peptide synthesis was performed using N^α^-Fmoc-protected amino acids, a HBTU-HOBt activation protocol, and a Tentagel resin with Rink amide linker (0.23 mmol/g); HBTU (3.8 eq.), HOBt-HOAt (4∶1, 4 eq.), Fmoc-AA-OH (4 eq.), DIPEA (7.2 eq.) in NMP. Manual peptide synthesiswas performed with preactivation for 5 min and single couplings for 90 min. Fmoc deprotections were performed using piperidine/NMP (1∶4) for 2+15 min.

Automated peptide synthesis was performed on a Biotage SyroWave. Standard Fmoc-amino acids were coupled in parallel mode 120 min: Fmoc-AA-OH (s5.2 eq.), HBTU (5 eq.), HOBt/HOAt (4∶1, 5 eq.), DIPEA (9.8 eq.). Arginine mimics were coupled at 75°C for 10 min: Fmoc-AA-OH (2 eq.), HBTU (1.9 eq.), HOBt/HOAt (4∶1, 2 eq.), DIPEA (3.6 eq.). Fmoc deprotections were performed using piperidine/NMP (2∶3) for 3 followed by piperidine/NMP (1∶4) for 15 min.

Peptides with Alloc protected amino acids were deprotected to a free amine by treating the fully protected and N-acetylated peptides with a mixture of Pd(PPh_3_)_4_ (0.05 eq.) and Me_2_NH⋅BH_3_ (0.2 eq.) in degassed CH_2_Cl_2_ (30 min) and washed with NMP (5 x). The peptides were then treated with *N*, *N*'-di-Boc-1H-pyrazole-1-carboxamidine (5 eq.) in NMP overnight. Following peptide assembly, the resins were washed extensively with NMP and CH_2_Cl_2_, before peptide release with TFA/H_2_O/triethylsilane (95∶2.5∶2.5). Peptide release proceeded for 2 h before the TFA-peptide mixture was collected by filtration. The resin was additionally washed with TFA (2x) and the TFA mixtures were pooled. TFA was removed under a stream of nitrogen and the peptide was precipitated with diethyl ether. The peptides were dissolved in a minimum amount of H_2_O/acetonitrile (2∶1) before being purified by preparative HPLC. The purified peptides were dissolved in H_2_O/acetonitrile (2∶1) to a final concentration of 1 mM. The solution was brought to pH 7.5–8 with NH_3_ in methanol. The peptides were oxidized to form disulfide bridges by addition of 1.2 eq. of H_2_O_2_ (30–60 min). The oxidization was stopped with the addition of acetic acid (0.1 ml) followed by evaporation and HPLC purification. Mass spectrometry: Mupain-1-12 D9A [M+H]^+^ 1224,4 [M+2H]^2+^ 613,1; mupain-1-16 D9A [M+H]^+^ 1234,5 [M+2H]^2+^ 617,7.

The concentrations of the peptide variants were determined by measurements of OD_280_ and the use of sequence-derived extinction coefficients provided by the Protparam tool on the Expasy server (located at http://www.expasy.org).

### Proteases

cDNA encoding full length muPA, full length huPA and site directed mutants were cloned into the pTT5 or pCDNA3.1 vectors. All variants contained a C-terminal hexa-His tag. The cDNAs were transfected into human embryonic kidney 293 (HEK293) 6E suspension cells, which were cultured in a humidified 5% CO_2_ incubator at 37°C. The medium used was F17 medium (Invitrogen) supplemented with 0.1% Pluronic F-68, a nonionic detergent (Invitrogen), 4 mM L-Gln (Lonza), and 25 µg/ml of the selective agent for eukaryotic cells G418 (Invitrogen). *M*_r_∼25,000 linear polyethylenimine (400 µg) (Polysciences) was preincubated with cDNA (200 µg) for 15 min and added to 200 mL cells with a density of 1×10^6^ cells/mL. Twenty-four hours post-transfection, Tryptone N1 (Organotechnie SAS) was added to a final concentration of 0.5% (w/v). Conditioned medium was collected 96 h post-transfection, and the recombinant proteins were purified using immobilised metal ion affinity chromatography followed by benzamidine-Sepharose affinity chromatography. The purified proteins were at least 95% pure, as judged by Coomassie Blue-stained SDS-PAGE gels. To ensure that the uPAs purified from the conditioned media were completely in the two-chain form, they were treated with plasmin for 2 hours in a 1∶100 ratio.

The cloning, production, and purification of recombinant uPA catalytic domain (residues 159–411), harbouring a H99Y mutation, to be used for crystallisation and isothermal titration calorimetry (ITC), was largely as described previously [16]. Basically, the recombinant catalytic domain of huPA-H99Y was secreted from a stable *Pichia pastoris* strain (X-33) after induction by methanol and captured by a cation exchange column. The protein was further purified on a gel filtration column (Superdex 75 HR 10/30 column from GE Health Care) equilibrated with 20 mM sodium phosphate, pH 6.5, 150 mM NaCl. The protein was eluted as a single peak under these conditions, with a retention time of approximately 13.6 ml. The recombinant uPA catalytic domain expressed in this way is an active protease with an activity comparable to full-length two-chain uPA [16]. The protein was dialysed in 20 mM potassium phosphate, pH 6.5 overnight and concentrated to 10 mg/ml, using stirred ultrafiltration cells (Millipore and Amicon Bioseparations, Model-5124), prior to protein crystallization. The recombinant catalytic domain of huPA-H99Y to be used for ITC assays was further purified with benzamidine-Sepharose affinity chromatography.

### Crystallization and data collection of uPA or uPA H99Y in complex with mupain-1 variants

The crystallization trials were carried out with the sitting-drop vapour-diffusion method. The crystals of the catalytic domain of huPA-H99Y were obtained by equilibrating huPA-H99Y protein against a reservoir solution containing 2.0 M ammonium sulfate, 50 mM sodium citrate, pH 4.6, and 5% polyethylene glycol (PEG) 400 at room temperature. The crystals appeared in about 3 days. The crystals of huPA-H99Y were then soaked for 2 weeks in new soaking buffer (40% PEG 4000, 0.1 M Tris-HCl, pH 7.4), containing 1 mM mupain-1 variants. A solution of 20% PEG 4000, 0.1 M Tris-HCl, pH 7.4 and 20% (v/v) glycerol was used as cryoprotectant for X-ray diffraction data of the crystals at the BL17U beamline, Shanghai Synchrotron Radiation Facility and 3W1A beamline, Beijing Synchrotron Radiation Facility (BSRF). The diffraction data was indexed and integrated by HKL2000 program package [17].

### Crystal structure determination and refinement

The crystal structures of the different complexes were solved by molecular replacement [18], using the uPA structure (PDB code: 2NWN) [16] as the search model. The electron density for the peptide was clearly visible in the uPA active sites and was modelled based on the F_o_–F_c_ difference map. The structures was refined (ccp4 program package)[18] and manually adjusted (by the molecular graphics program COOT) [19] iteratively until the convergence of the refinement. Solvent molecules were added using a F_o_–F_c_ Fourier difference map at 2.5 σ in the final refinement step. Statistics of data collection and final model refinement are summarized in Supporting Table S1. The final structure was analysed by software Pymol [20].

### Model of mouse uPA

The sequence of the catalytic domain of muPA (positions 16–243) (Uniprot P06869, EC: 3.4.21.73) was homology modeled onto the X-ray crystal structure of huPA-H99Y, using Molecular Operating Environment [21]. The sequence identity is 71% between muPA and huPA-H99Y (S1 Fig.). The generated molecular model was refined by CNS program package [22].

### Determination of *K*_M_ values

To determine the *K*_M_ values for hydrolysis of S-2444 (pyro-Glu-Gly-Arg-*p*-nitroanilide) by the different uPA variants used in the present study, a 200 µL 2-fold dilution series of the substrate (4 - 0 mM for huPA variants, 24 – 0 mM for muPA variants) in a buffer of 10 mM HEPES, pH 7.4, 140 mM NaCl (HEPES-buffered saline, HBS), with 0.1% bovine serum albumin (BSA), was incubated 2 min at 37°C, prior to the addition of a fixed concentration of each protease (approximaterly 2 nM final concentration). The initial reaction velocities (*V*_i_), monitored as the changes in absorbance at 405 nm, were plotted against the initial substrate concentration ([S]) and non-linear regression analysis was used to determine the *K*_M_ according to equation 1: 



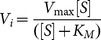
(1)


The *K*_M_ values for hydrolysis of S-2444 by the uPA variants employed in the present study are listed in S2 Table.

### Determination of *K_i_* values

For routine determination of *K*_i_ values for the inhibition of the various enzymes under steady state inhibition conditions, a fixed concentration of purified enzyme or conditioned media from transfected cells (approximately 2 nM enzyme as the final concentration) was pre-incubated in a total volume of 200 µL HBS with 0.1% BSA at 37°C, with various concentrations of mupain-1 variants for 15 min prior to the addition of the chromogenic substrate in concentrations approximately equal to the *K*_M_ value for each particular variant. The initial reaction velocities were monitored at an absorbance of 405 nm. The inhibition constants (*K*_i_) were subsequently determined from the non-linear regression analyses of plots for *V*_i_/*V*_o_ versus [I]_0_, using Equation 2, derived under assumption of competitive inhibition:



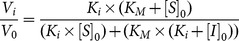
(2)where *V*_i_ and *V*_0_ are the reaction velocities in the presence and absence of inhibitor, respectively; [S]_0_ and [I]_0_ are the substrate and inhibitor concentrations, respectively; *K*_M_ is *K*_M_ for substrate hydrolysis by each protease. In Equation 2, it is assumed that [*S*]_free_ ≈ [*S*]_0_ and [I]_free_ ≈ [*I*]_0_. These conditions were fulfilled, as less than 10% of the substrate was converted to product in the assays and as the assay typically contained a final concentration of each protease of 2 nM and inhibitor concentrations in the µM range.

In cases, in which we observed no measurable inhibition (*i.e*., <10%) at the maximal inhibitor concentration used, *i.e*., 400 µM, the accuracy of the assay allowed us to conclude that the *K*_i_ value was more than 1000 µM (indicated as “>1000 µM” in the tables).

The validity of performing the *K*_i_ determinations with uPA-containing conditioned media from transfected cells was verified by controls in which the determinations were performed with conditioned media as well as with purified preparations. These controls were performed with murine uPA wt and human uPA wt, obtaining indistinguishable values with the two types of samples [13].

### Surface plasmon resonance (SPR) analysis

To determine the equilibrium binding constants (*K_D_*), the association rate constants (*k_on_*) and dissociation rate constants (*k_off_*) for peptide binding to uPA, surface plasmon resonance analysis was performed on a Biacore T200 instrument (Biacore, Uppsala, Sweden). A CM5 chip was coupled with the uPA variant (muPA or huPAH99Y) to be analysed, by injecting a concentration of 30 µg/mL uPA in immobilization buffer (10 mM sodium acetate, pH 5.0), aiming for an immobilised level of approximately 500 response units (RU). Immobilisation was followed by surface blocking with ethanolamine. A reference cell was prepared in the same way, without coupling of uPA. Mupain-1 variants in HBS with 0.1% BSA, in a dilution series, were injected at a flow rate of 30 µL per min during 60 s at 25°C. Subsequently, the dissociation was monitored during 600 s. Kinetic constants (*k*_on_ and *k*_off_) were calculated with the Biacore Evaluation Software, using the 1∶1 kinetic fit. The *K*_D_ values were calculated as *k*_off_ */k*_on_.

### ITC

For these experiments, we used the catalytic domain of huPA-H99Y expressed in and purified from *Pichia pastoris* strain X-33 (see above). The protein was dissolved in and dialysed against a buffer of 20 mM sodium phosphate, pH 7.4, 140 mM NaCl. The protein concentration was determined by absorbance at 280 nm, using an extinction coefficient of 43810 M^−1^cm^−1^. The peptides were dissolved in the above-mentioned buffer. All isothermal titration calorimetry experiments were performed with a MicroCal^TM^ ITC200 instrument equilibrated to a temperature of 25°C (298oK). The concentration of uPA-H99Y catalytic domain used in the 200 µl sample cell was 5–50 µM, depending on the affinity of the ligand. Titrations were performed by injecting 2 µl of the ligand until the total syringe volume of 40 µl was spent. Titration of ligand into buffer was performed to obtain buffer correction. The equilibrium association constant KA and the reaction enthalpy ΔH were calculated by fitting the integrated titration peaks using a one-binding-site model in the ITC ORIGIN7 programme package. The following formulas for Gibbs free energy ΔG were used to analyse the measured energies



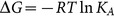
(3)




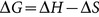
(4)where R is the gas constant and T the absolute temperature. ΔS, the entropic change during the reaction, was calculated using equations 3 and 4 and the measured *K*_A_ and ΔH values.

### NMR spectroscopy

Peptide samples were dissolved in a buffer of 10 mM sodium phosphate, 140 mM NaCl in D_2_O/H_2_O (7∶93, v/v). The pH was adjusted to 7.4. For chemical-shift reference and to increase the long-term stability of the samples, 2,2-dimethyl-2-silapentane-5-sulfonic acid (10 µM) and NaN_3_ (150 µM) were added. The peptide concentrations were 5.0 and 6.7 mM for mupain-1 and mupain-1-16, respectively. NMR experiments were acquired with a Bruker Avance III 500 spectrometer (500.13 MHz; Bruker Biospin, Rheinstetten) equipped with a standard inverse triple-resonance TXI 5 mm probe. Two-dimensional TOCSY data (80 ms mixing time), NOESY data (200 ms mixing time) and natural abundant ^13^C HSQC data were acquired for both peptides. The experiments were acquired at 5°C to slow down peptide tumbling, favour lowest-energy conformations, and obtain the highest signal in the NOESY spectra for assignment. Assignments were obtained by standard methods with CCPN software [23]. Visualisation of spectra and integration of NOE peaks were performed in SPARKY [24]. Random coil shifts were calculated by using values provided by Kjærgaard et al. [25] and corrected by subtraction of correction values from Schwarzinger [26] which contains values for oxidised Cys and for cis-Pro. These correction values were obtained by subtracting Schwartzinger's values for Cys_red_ from Cys_ox_ and trans-Pro from cis-Pro for all the different proton types in these residues. The order parameter (S^2^) was calculated according to the method of Berjanskii and Wishart [27] as implemented within TALOS+ [28].

## Results

### Inhibitory mechanism and binding mode studied by X-ray crystal structure analysis

**Figure 2:**
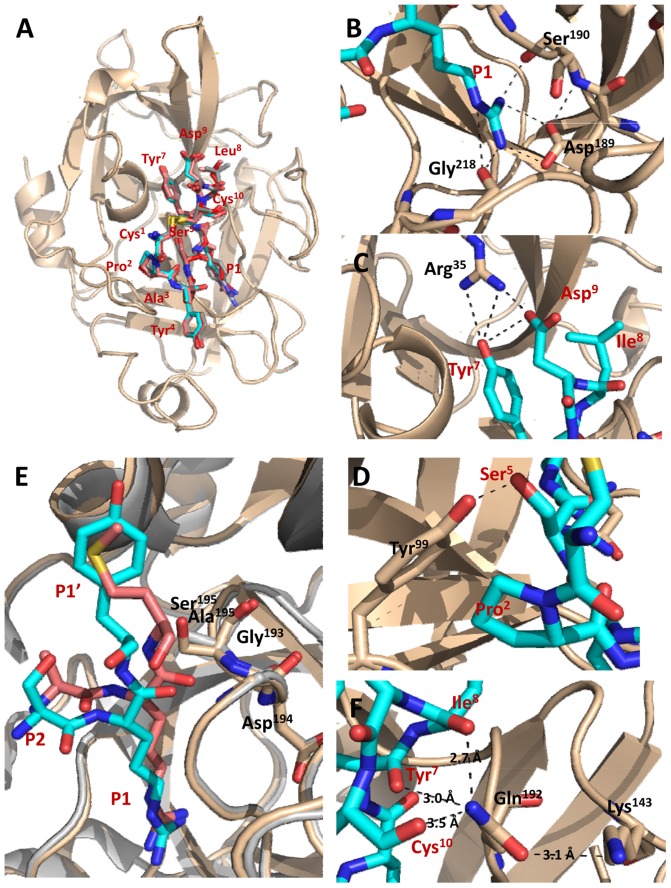
X-ray crystal structure analysis of huPA-H99Y in complex with peptidic inhibitors. (A) Overall structure of the complexes between huPA-H99Y and mupain-1 (cyan), mupain-1-12 (salmon), mupain-1-16 (grey), and mupain-1-16-D9A (red). (B) A zoom on interactions of mupain-1's Arg^6^ in the S1 pocket; polar interactions are indicated by stippled lines. (C) A zoom on the polar interactions (stippled lines) between huPA-H99Y residue Arg^35^ and mupain-1 residues Tyr^7^ and Asp^9^. (D) A zoom on the polar interaction (stippled lines) between huPA-H99Y residue Tyr^99^ and mupain-1 residue Ser^5^. (E) An overlay of the active site areas of the huPA-H99Y – mupain-1 complex and the huPA S195A – PAI-1 Michaëlis complex (pdb entry 3pb1; [29]); the P2, P1, and P1′ residues are indicated, those of PAI-1 in salmon. (F) A zoom on the Lys^143^ – Gln^192^ area of the huPA-H99Y – mupain-1 complex; distances, in Å, between different residues are indicated. In all parts of the figure, huPA-H99Y are shown in wheat cartoon presentation. In Fig. 1E, huPA S195A is shown in grey cartoon presentation. The peptides are shown in stick representation. huPA-H99Y residues are labelled with black letters, peptide residues with dark red letters.

While being unable to generate crystals of muPA, we did manage to crystallise huPA-H99Y, a murinised version of human uPA which, in contrast to human uPA wt, is able to bind mupain-1, although with a somewhat lower affinity than muPA [12], [13]. We determined the structures huPA-H99Y in complex with mupain-1 (CPAYSRYLDC) itself as well as with either of two other inhibitory peptides, namely mupain-1-12 (CPAYS[4-guanidinophenylalanine]YLDC) and mupain-1-16 (CPAYS[L-3-(N-amidino-4-piperidyl)alanine]YLDC). Mupain-1-12 and mupain-1-16 have around 10-fold higher affinities to huPA-H99Y than mupain-1 [13]. X-ray data collection and model refinement statistics are shown in S1 Table. Important features of the structures are illustrated in Fig. 2. The contact distances between the residues of the peptides and residues of huPA-H99Y are shown in S3-S5 Tables. The B-factors are listed in S7 Table.

The analysis showed very similar conformations of the three peptides when bound to huPA-H99Y (Fig. 2A). The RMSD values among these peptides are quite small (0.32–0.35 Å). In the complexes, the inhibitory peptides adopt cyclic conformations with an overall Ω shape. The disulfide bonds are the main structural restraint responsible for this conformation. Beginning from the N-terminus, the cyclic peptides approach the active site of huPA-H99Y from the 99-loop, insert residue 6 into the S1 pocket, and exit the active site towards the 37-loop (Fig. 2A). In each of the structures, the amino acid in position 6 of the peptide, *i.e*., Arg, 4-guanidinophenylalanine, or L-3-(N-amidino-4-piperidyl)alanine, forms polar interactions to Asp^189^, Ser^190^, and Gly^218^ in the S1 pocket (Fig. 1B). In addition, the X-ray crystal structure analysis showed that the huPA-H99Y residues Arg^35^, Val^41^, Leu^97b^, Tyr^99^, Gln^192^, Trp^215^, and Arg^217^ have the largest contact surface area to the peptides (S3-S5 Tables). In particular, Arg^35^ forms polar interactions with peptide residues Tyr^7^ and Asp^9^ (Fig. 2C) and Tyr^99^ forms polar interactions with peptide residue Ser^4^ (Fig. 2D). In the enzyme-peptide structures, the peptides are constrained by two type I tight β-turns (Pro^2^-Ala^3^-Tyr^4^-Ser^5^ and Tyr^7^-Leu^8^-Asp^9^-Cys^10^) and three intra-peptide H-bonds (Pro^2^ O – Ser^5^ N; Ser^i5^ Oγ - Tyr^7^ N; Ser^5^ Oγ - Arg^6^ N; S2 Fig.). The tight β-turns are likely to play an important role in maintaining the conformation and stability of the bound peptide. On the enzyme side, there are no major changes in the conformation of surface loops of the enzyme following peptide binding based on the comparison of the structure of the enzyme in the absence or the presence of peptide.

The inhibitory mechanism of these peptides and the reason that they are inhibitors and not substrates readily become evident from the structural analysis. The overall conformation of the peptide backbone on the enzyme surface is quite similar to that of the reactive centre loop of plasminogen activator inhibitor-1 (PAI-1) in its Michaëlis complex with uPA S195A, aligning into the active site in a substrate-like manner (Fig. 2E; [29]). However, compared to PAI-1 in its Michaëlis complex with uPA S195A, the scissile bond of mupain-1 in its complex with uPA-H99Y is shifted approximately 0.5 Å away from residue 195. The distance from the Ser^195^ Oγ to the carbonyl group of the P1 residue of mupain-1 and derivatives is too large (approximately 3.9 Å) to allow the nucleophilic attack associated with catalysis (Fig. 2E). Moreover, the distance from the oxygen atom of the carbonyl group of the P1 residue to the amido group of the Ser^195^ is too large (approximately 4 Å) for formation of a polar interaction, implying that oxyanion stabilisation cannot take place (Fig. 2E).

In spite of the X-ray crystal structure analysis having a good resolution, no differences could be detected between the complexes of huPA-H99Y with each of the peptides mupain-1, mupain-1-12, and mupain-1-16, the *K*_i_ values of which differ around 10-fold. A 10-fold difference in *K*_i_ corresponds to a Δ(ΔG) for binding of approximately 6 kJ/mol, about the same energy as that of an average hydrogen bond. In this case, it is therefore possible that small differences in hydrophobic interactions and in the length and angles of polar interactions, at the detection limit by the structural analysis, may account for the differences in *K*_i_ values. Alternatively, and more likely, the peptide-enzyme complexes, in solution, may sample a number of similar conformations of which only the most stable one is selected during crystallisation.

### Analysis of peptide-huPA-H99Y exosite interactions by site-directed mutagenesis

We determined the *K*_i_ values for inhibition of variants of huPA-H99Y by variants of mupain-1, mupain-1-12, and mupain-1-16. The Ala-substituted residues of the peptide and of huPA-H99Y are those deemed to be important for binding from the X-ray crystal structure analysis (Table 1). In good agreement with the structural analysis, Ala-substitution of mupain-1 residues Pro^2^, Tyr^4^, Ser^5^, and Arg^6^ led to strong reductions in affinity, Ala-substitution of Tyr^7^ to a moderate reduction in affinity, and Ala-substitution of Leu^8^ to a very small change in affinity. The observed effect of Ala-substituting the peptide's Ser^5^ is in good agreement with the fact that the binding of mupain-1 to huPA is dependent on the substitution of its His^99^ with Tyr [12], shown here to be able to form a hydrogen bond with Ser^5^ (Fig. 2D). Surprisingly, Ala-substitution of Asp^9^ of mupain-1, mupain-1-12, and mupain-1-16 led to a 3–10-fold increased affinity to huPA-H99Y. This observation is in contrast to the expectancies from the X-ray crystal structure analysis, which predicts a polar interaction between Asp^9^ of mupain-1 and Arg^35^ of huPA-H99Y (Fig. 2C; S3-S5 Tables).

**Table 1:** Inhibition of huPA-H99Y and huPA-H99Y exosite mutants by mupain-1 variants.

Peptide name	Sequence	huPA-H99Y	huPA-H99Y-R35A	huPA-H99Y-V41A	huPA-H99Y-K143A	huPA-H99Y-Q192A
Mupain-1	CPAYSRYLDC	15.3±2.0 (3)*	39.5±2.9 (3)	32.4±5.3 (3)	56.6±8.0 (3)	33.2±4.2 (3)
Mupain-1 P2A	CAAYSRYLDC	>1000	nd	nd	nd	nd
Mupain-1 Y4A	CPAASRYLDC	>1000	nd	nd	nd	nd
Mupain-1 S5A	CPAYARYLDC	>1000	nd	nd	nd	nd
Mupain-1 R6A	CPAYSAYLDC	>1000	nd	nd	nd	nd
Mupain-1 Y7A	CPAYSRALDC	44.0±3.0 (3)	nd	nd	nd	nd
Mupain-1 L8A	CPAYSRYLDC	19.1±1.1 (3)	nd	nd	nd	nd
Mupain-1 D9A	CPAYSRYLAC	5.99±0.43 (3)	2.68±0.29 (3)	7.20±1.44 (3)	8.74±0.64 (3)	4.85±0.32 (3)
Mupain-1-12	CPAYS[4-guanidino-phenyl-alanine]YLDC	1.86±0.74 (3)*	3.26±1.04 (3)	1.60±0.54 (3)	2.14±0.78 (3)	2.35±0.74 (3)
Mupain-1-12 D9A	CPAYS[4-guanidino-phenyl-alanine]YLAC	0.186±0.036 (3)	0.231±0.078 (3)	0.478±0.137 (3)	0.402±0.146 (1)	0.438±0.079 (3)
Mupain-1-16	CPAYS[L-3-(N-amidino-4-piperidyl)alanine]YLDC	2.48±0.07 (3)*	2.50±0.29 (3)	1.95±0.30 (3)	1.13±0.32 (3)	2.71±0.28 (3)
Mupain-1-16 D9A	CPAYS[L-3-(N-amidino-4-piperidyl)alanine]YLAC	0.309±0.013 (3)	0.174±0.059 (3)	0.480±0.158 (3)	0.188±0.057 (3)	0.438±0.072 (4)

The *K*_i_ values (in µM) for inhibition of the indicated enzymes by the indicated peptides at 37°C are shown as means ± S.D; the numbers of determinations are indicated in parentheses. *These values are reproduced from previous publications [Andersen et al., 2008; Hosseini et al., 2011] and shown here to facilitate comparison.

Ala-substitution of huPA-H99Y residues Arg^35^, Val^41^, Lys^143^, and Gln^192^ led to 2–4 fold reductions in affinity to mupain-1, but smaller if any reductions in the affinity to mupain-1-D9A, mupain-1-12, mupain-1-16, mupain-1-12-D9A, and mupain-1-16-D9A. The effects of the R35A and Q192A mutations are in good agreement with the predictions of polar interactions from the X-ray crystal structure analysis (Fig. 2C, Fig. 2F). The V41A mutation may result in loss of hydrophobic interactions. From the X-ray crystal structure analysis, Lys143 is not predicted to make any direct contacts to the peptides, but the observed effect of the K143A mutation may be caused by an indirect effect through a polar interaction between Lys 143 and Gln192 (Fig. 2F). The observed changes following the Ala substitutions in the enzyme were in all cases small, in agreement with the fact that the predicted polar interactions are surface exposed. In general, there was less dependence on the exosite interactions with the peptides with the unnatural P1 residues and the peptides with an D9A substitution. This observation suggests that the effects of the exosite mutations and the D9A substitution is influenced by interactions in the S1 pocket.

The *K*_i_ determinations were supported by determinations of *K*_D_ values with SPR (Table 2; S3 Fig.) and ITC (Table 3). The *K*_D_ values determined by SPR and ITC agreed well with the *K*_i_ values, in particular when considering that *K*_D_ values were routinely determined at 25°C and the *K*_i_ values at 37°C; separate control experiments showed that the *K*_i_ values were 2–3 fold lower at 25°C than at 37°C (data not shown). The SPR measurements showed that the 9–12-fold increased affinities to huPA-H99Y following the D9A substitution were associated with 3–4-fold increased *k*_on_ values as well as 2–3-fold decreased *k*_off_ values (Table 2). The ITC measurements showed that the increased affinity of the Ala^9^ peptides to huPA-H99Y, as compared to the original Asp^9^ peptides, was associated with a binding entropy penalty but mainly accounted for by a more favourable binding enthalpy (except with mupain-1 and mupain-1 D9A, in which case the differences were not statistically significant; Table 3). The most ready molecular interpretation of the SPR and ITC data, taken together, is that the D9A substitution renders the peptide more flexible in solution, thereby making the binding entropy less favourable, the association activation energy lower, and the *k*_on_ higher, and allows a more favorable binding enthalpy and a more stable bound state, thereby increasing the dissociation activation energy and decreasing the *k*_off_.

**Table 2:** Surface plasmon resonance analysis of the binding of peptides to muPA or huPA-H99Y.

		huPA-H99Y			muPA		
Peptide name	Sequence	*k*_on_ (M^-1^s^-1^), ×10^-5^	*k*_off_ (s^-1^), ×10^2^	*K*_D_ (µM)	*k*_on_ (M^−1^s^−1^), ×10^−5^	*k*_off_ (s^−1^), ×10^2^	*K*_D_ (µM)
Mupain-1	CPAYSRYLDC	0.569±0.103 (4)	80.2±7.6 (4)	14.1±2.4 (4)	0.914±0.250 (6)	3.64±1.03 (6)	0.398±0.046 (6)
Mupain-1 D9A	CPAYSRYLAC	2.60±0.20 (3)	36.6±0.21 (3)	1.41±0.11 (3)	0.985±0.169 (3)	4.01±0.31 (3)	0.417±0.091 (3)
Mupain-1-12	CPAYS[4-guanidino-phenyl-alanine]YLDC	2.06±0.15 (4)	24.6±1.2 (4)	1.21±0.13 (4)	1.56±0.95 (4)	1.92±0.63 (4)	0.138±0.034 (4)
Mupain-1-12 D9A	CPAYS[4-guanidino-phenyl-alanine]YLAC	5.93±0.33 (3)	7.94±0.39 (3)	0.134±0.002 (3)	2.42±1.01 (3)	3.94±0.30 (3)	0.177±0.051 (3)
Mupain-1-16	CPAYS[L-3-(N-amidino-4-piperidyl)alanine]YLDC	1.07±0.15 (4)	8.93±0.46 (4)	0.844±0.132 (4)	0.898±0.545 (3)	0.671±0.119 (3)	0.0894±0.0376 (3)
Mupain-1-16 D9A	CPAYS[L-3-(N-amidino-4-piperidyl)alanine]YLAC	4.65±0.27 (4)	3.33±0.13 (4)	0.0718±0.006 (4)	5.79±0.82 (3)	1.22±0.06 (3)	0.0213±0.0024 (3)

The table shows the rate constants and the *K*_D_ values for the binding of the indicated peptides to muPA or huPA-H99Y at 25°C, pH 7.4. Means, standard deviations, and numbers of determinations are indicated. Examples of sensorgrams are shown in S3 Fig.

**Table 3:** Isothermal titration calorimetry for binding of peptides to huPA-H99Y.

Peptide name	Peptide sequence	N	*K*_D_ (µM)	ΔG (kJ/mole)	ΔH (kJ/mole)	TΔS (kJ/mole)
Mupain-1	CPAYSRYLDC	1.09±0.15 (4)	5.04±1.61 (4)	−30.4±0.7 (4)	−36.5±3.2 (4)	−6.1±2.6 (4)
Mupain-1 D9A	CPAYSRYLAC	1.03±0.09 (6)	1.17±0.30 (6)^1^	−33.9±0.7 (6)^1^	−37.9±2.4 (6)	−4.0±2.9 (6)
Mupain-1-12	CPAYS[4-guanidino-phenylalanine]YLDC	0.85±0.08 (4)	0.400±0.016 (4)	−36.5±0.1 (4)	−43.8±5.5 (4)	−7.6±5.6 (4)
Mupain-1-12 D9A	CPAYS[4-guanidino-phenylalanine]YLAC	0.88±0.01 (3)	0.138±0.047 (3)^1^	−39.2±0.8 (3)^1^	−60.1±5.4 (3)^1^	−18.9±5.1 (3)^1^
Mupain-1-16	CPAYS[L-3-(N-amidino-4-piperidyl)alanine]YLDC	0.87±0.09 (4)	0.380±0.065 (4)	−36.7±0.4 (4)	−33.2±4.2 (4)	3.5±1.0 (4)
Mupain-1-16 D9A	CPAYS[L-3-(N-amidino-4-piperidyl)alanine]YLAC	0.88±0.11 (5)	0.134±0.061 (5)^1^	−39.5±1.1 (5)^1^	−46.6±2.9 (5)^1^	−6.2±2.7 (5)^1^

1 The value for the D9A peptide is significantly different from the value for the unmodified peptide (p<0.01).

The table shows thermodynamic parameters for the binding of the indicated peptides to huPA-H99Y at 25°C, pH 7.4. Means, standard deviations, and numbers of determinations are indicated.

Considering the unexpected effect of the D9A substitution, we also crystallised mupain-1-16 D9A in complex with huPA-H99Y (S1 and S6 Tables), but in spite of the much higher affinity of the D9A peptides, the structures of the mupain-1-16 and the mupain-1-16-D9A complex were indistinguishable (Fig. 2A). Noticeably, however, the relative B-factor for mupain-1-16 D9A was higher than that for mupain-1-16 (1.39 versus 1.24; S7 Table).

In summary, while most interactions between the peptides and huPA-H99Y predicted by X-ray crystal structure analysis were largely in agreement with the results of the site-directed mutagenesis, the increased affinity following the D9A substitution in mupain-1 was unexpected and could not be correlated with any structural differences observable by X-ray rystal structuyre analysis. However, the SPR and ITC analyses indicated a more stable complex following the D9A substitution, in spite of a binding entropy penalty.

### Analysis of peptide-muPA exosite interactions by site-directed mutagenesis

**Figure 3:**
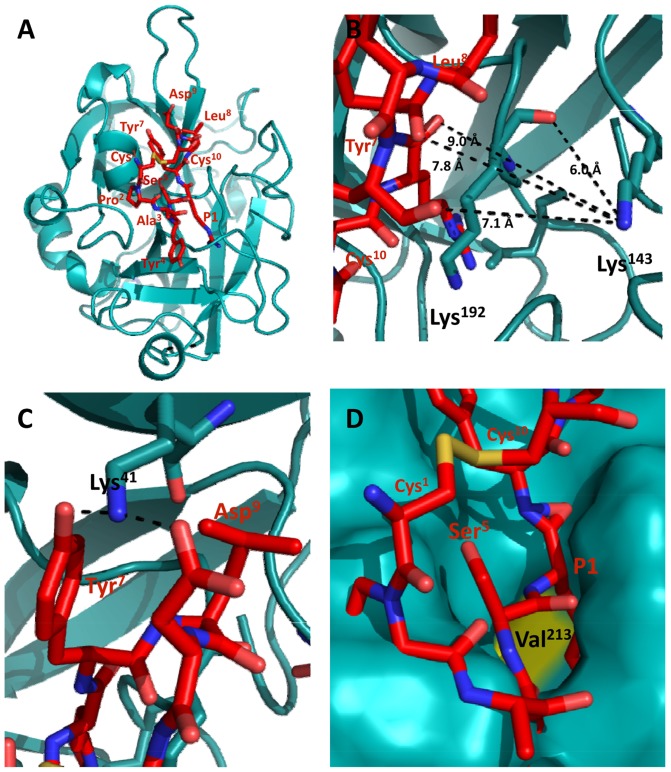
Structural model of mupain-1 in complex with muPA. (A) Overall model of the complex between muPA (teal ribbon presentation) and mupain-1 (red stick representation). The enzyme is shown in teal cartoon presentation. (B) A zoom on the Lys^143^ area of the model, showing distances from Lys^143^ to the closest atoms in the peptide. (C) A zoom on the Lys^41^ area of the model, showing the distances between Lys41 and peptide residues Tyr^7^and Asp^9^. (D) A zoom on the entrance to the S1 pocket of the model, with Val^213^ at the entrance indicated; the enzyme is represented as teal surface. huPA-H99Y residues are labelled with black letters, peptide residues with dark red letters.

Next, we analysed the *K*_i_ values for inhibition of several muPA variants by mupain-1, mupain-1-12, and mupain-1-16, Ala substituting residues of the peptide and the enzyme in positions implicated in peptide-enzyme interaction by the X-ray crystal structure analysis of the huPA-H99Y-peptide complex (see S1 Fig. for alignment of the amino acid sequences of the catalytic domains of huPA and muPA). Previously, we showed that Ala-substitution of mupain-1 residues Pro^2^, Tyr^4^, Ser^5^, Arg^6^, and Tyr^7^ leads to substantial loss of affinity to muPA, while Leu^8^ and Asp^9^ could be Ala-substituted with minimal consequences [12]. Thus, the effect of the D9A substitution is different with muPA and huPA-H99Y. Now, we found that Ala-substitutions of muPA residues Lys^41^, Tyr^99^ and Lys^143^ increase the *K*_i_ values 5–50-fold (Table 4). However, in contrast to observations with huPA-H99Y, there were no effects of Ala substitutions of muPA residues in the 37-loop. From a model of muPA in complex with mupain-1, it seems that Lys^41^ is able to make polar interactions with Tyr^7^ (2.3 Å) and Asp^9^ (2.8 Å) of mupain-1 (Fig. 3C), explaining the effect of substitution of this muPA residue. From the model, Lys^143^ is predicted to be more than 6 Å away from the closest part of the peptide and more than 6 Å away from Lys^192^ (Fig. 3B), leading to the notion that the peptide and/or residue 192 have different conformations in the huPA-H99Y-mupain-1 complex and the muPA-mupain-1 complex. Thus, the observed importance of mupain-1 residues Pro^2^, Tyr^4^, Ser^5^ and Arg^6^ and muPA residue Tyr^99^ suggests that the CCPAYSR stretch of peptides occupies a position on muPA similar to that inferred from the X-ray crystal structure analysis of the peptide-huPA-H99Y complexes, while the stretch YLD of mupain-1 makes different interactions in the two complexes.

**Table 4:** Inhibition of muPA wt and muPA exosite mutants by mupain-1 variants.

Peptide name	Sequence	muPA-wt	muPA-K41A	muPA-Y99A	muPA-K143A
Mupain-1	CPAYSRYLDC	0.550±0.080 (8)*	4.29±0.15 (3)	26.0±5.8 (3)	4.88±0.35 (3)
Mupain-1 D9A	CPAYSRYLAC	0.890±0.020 (4)*	0.270±0.029 (3)	27.2±3.3 (3)	4.34±0.64 (3)
Mupain-1-12	CPAYS[4-guanidino-phenyl-alanine]YLDC	0.280±0.020 (5)*	1.72±0.24 (3)	5.20±0.06 (3)	1.00±0.04 (3)
Mupain-1-12 D9A	CPAYS[4-guanidino-phenyl-alanine]YLAC	0.190±0.010 (3)	0.091±0.007 (3)	5.89±1.31 (3)	1.00±0.03 (3)
Mupain-1-16	CPAYS[L-3-(N-amidino-4-piperidyl)alanine]YLDC	0.045±0.010 (4)*	0.30±0.05 (3)	1.24±0.44 (3)	0.13±0.02 (3)
Mupain-1-16 D9A	CPAYS[L-3-(N-amidino-4-piperidyl)alanine]YLAC	0.076±0.003 (3)	0.014±0.002 (3)	1.36±0.33 (3)	0.10±0.02 (3)

The *K*_i_ values (in µM) for inhibition of the indicated enzymes by the indicated peptides are shown as means ± S.D; the numbers of determinations are indicated in parentheses. *These values are reproduced from previous publications [Andersen et al., 2008; Hosseini et al., 2011] and shown here to facilitate comparison.

Besides the muPA mutants reported in the table, we found no significant change in *K*_i_ values after the following substitutions: Q35A; N37A; K37aA; G37cA; S37dA; P37eA; P38A; Q60aA; E146A; Y149A. For unknown reasons, muPA K192A could not be expressed.

Interestingly, the D9A substitution, while having no effects on the affinities of the peptides to muPA wt, muPA-Y99A, and muPA-K143A, strongly increased the affinity to muPA-K41A (Table 2). Thus, the *K*_i_ values for inhibition of muPA by the Ala^9^ peptides depend differently on exosite mutations than those for the original Asp^9^ peptides. This observation shows than the interactions between the Ala^9^ peptides and muPA differ from the interactions between the Asp^9^ peptides and muPA. Importantly, the D9A substitution in all cases reduced the difference in *K*_i_ between huPA-H99Y and muPA (Tables 1 and 4): For the Asp^9^ peptides, the *K*_i_ value for inhibition of huPA-H99Y were 28, 7, and 55-fold higher than those for inhibition of muPA, while the corresponding values for the Ala^9^ peptides were only 7, 1, and 4-fold higher.

The *K*_i_ values were in good agreement with the *K*_D_ values determined by SPR (Table 2).

### Analysis of S1-P1 interactions by site-directed mutagenesis

In order to characterise the mechanism of the increase in affinity following substitution of the P1 Arg with either of the two unnatural P1 residues, we introduced mutations in the S1 pocket of muPA, *i.e*., S190A and V213T (Table 5). The X-ray crystal structure analysis of the peptide-huPA-H99Y complexes implicated Ser^190^ in hydrogen bonding to the P1 residues (Fig. 2B; S3–S5 Tables); many serine proteases, including tissue-type plasminogen activator (tPA), have an Ala in this position. Val^213^ forms a hydrophobic patch at the entrance to the S1 pocket (Fig. 3D); we substituted the Val with a Thr, as some serine proteases, including plasma kallikrein, has a Thr in this position. The S190A substitution resulted in a decreased affinity for all the peptides, while the V213T mutation resulted in an increased affinity for all the peptides (Table 5).

**Table 5:** Inhibition of muPA wt and muPA S1 pocket mutants by mupain-1 variants.

Peptide name	Sequence	muPA wt	muPA-S190A	muPA-V213T
Mupain-1	CPAYSRYLDC	0.550±0.080 (8)*	1.60±0.53 (3)	0.246±0.044 (3)
Mupain-1 D9A	CPAYSRYLAC	0.890±0.020 (4)*	3.02±0.33 (3)	0.373±0.055 (3)
Mupain-1-12	CPAYS[4-guanidino-phenyl-alanine]YLDC	0.280±0.020 (5)*	2.30±0.46 (3)	0.086±0.030 (3)
Mupain-1-12 D9A	CPAYS[4-guanidino-phenyl-alanine]YLAC	0.190±0.010 (3)	3.52±0.50 (3)	0.166±0.075 (4)
Mupain-1-16	CPAYS[L-3-(N-amidino-4-piperidyl)alanine]YLDC	0.045±0.010 (4)*	0.352±0.040 (3)	0.013±0.003 (3)
Mupain-1-16 D9A	CPAYS[L-3-(N-amidino-4-piperidyl)alanine]YLAC	0.076±0.003 (3)	0.529±0.052 (3)	0.014±0.004 (3)

The *K*_i_ values (in µM) for inhibition of the indicated enzymes by the indicated peptides are shown as means ± S.D; the numbers of determinations are indicated in parentheses. *These values are reproduced from previous publications [Andersen et al., 2008; Hosseini et al., 2011] and shown here to facilitate comparison.

**Figure 4:**
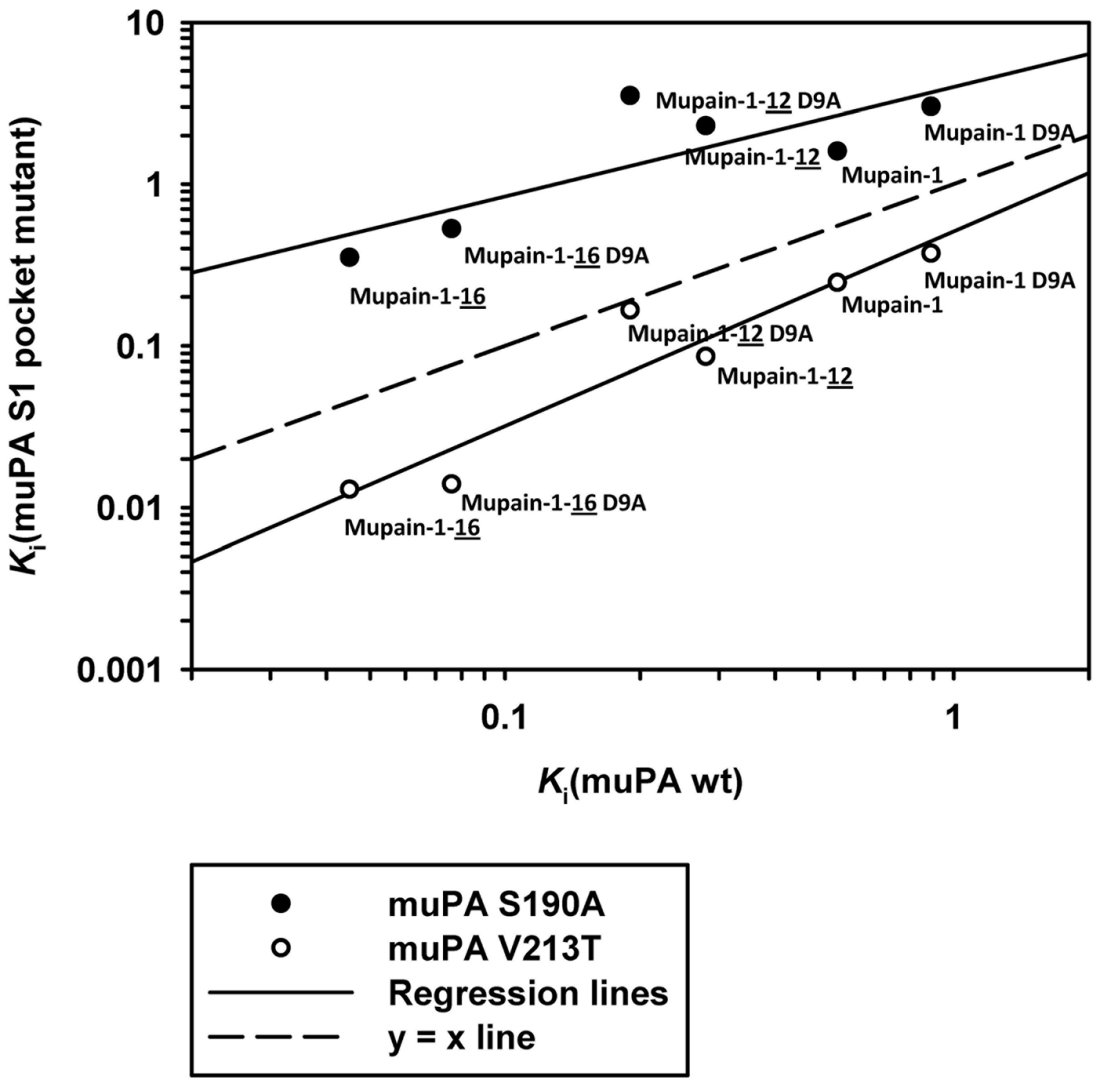
The relationship between the *K*_i_ values for inhibition of muPA S1 mutants and the *K*_i_ values for inhibition of muPA wt. The figure is based on the *K*_i_ values presented in Table 6. The x-axis shows the *K*_i_ values for inhibition of muPA wt by the indicated peptides. The y-axis shows the corresponding *K*_i_ values for inhibition of muPA S190A (filled dots) or muPA V213T (open dots). The lines resulted from simple linear regression analysis. The slopes of the lines are 0.68 (muPA-S190A) and 1.27 (V123T). The stippled line is the one which would have resulted if the *K*_i_ values for inhibition of the muPA S1 mutants had been identical to those for inhibition of muPA wt (y = x).

In order to visualise the effects of the S1 pocket mutations, the *K*_i_ values for the inhibition of the muPA mutants by each of the 6 peptides were plotted logarithmically *versus* the *K*_i_ values for inhibition of muPA wt by the same peptides (Fig. 4). For data points above the y = x line, the mutations increase the *K*_i_ value or decrease the affinity; for points above the y = x line, the mutations decrease the *K*_i_ value or increase the affinity. The slopes of the lines defined by the data points (which may be referred to as an “interdependence factor”) were 0.68 in the case of the S190A mutation and 1.27 in the case of the V213T mutation. The slopes being different from 1 shows that the effects of the S1 pocket mutations vary with the P1 residue: Following the S190A mutation, the *K*_i_ value increases more with mupain-1-12 and mupain-1-16 variants than with the Arg variants, while following the V213T mutation, the *K*_i_ value decreases more with the mupain-1-12 and mupain-1-16 variants than with the mupain-1 Arg variants. For instance, the V213T mutation increases the affinity for the mupain-1 Arg variants around 2.5-fold, but increases the affinity for the L-3-(N-amidino-4-piperidyl)alanine] variants around 5-fold. These findings are compatible with the notion that each of the three P1 residues fit differently into the S1 pocket.

**Figure 5:**
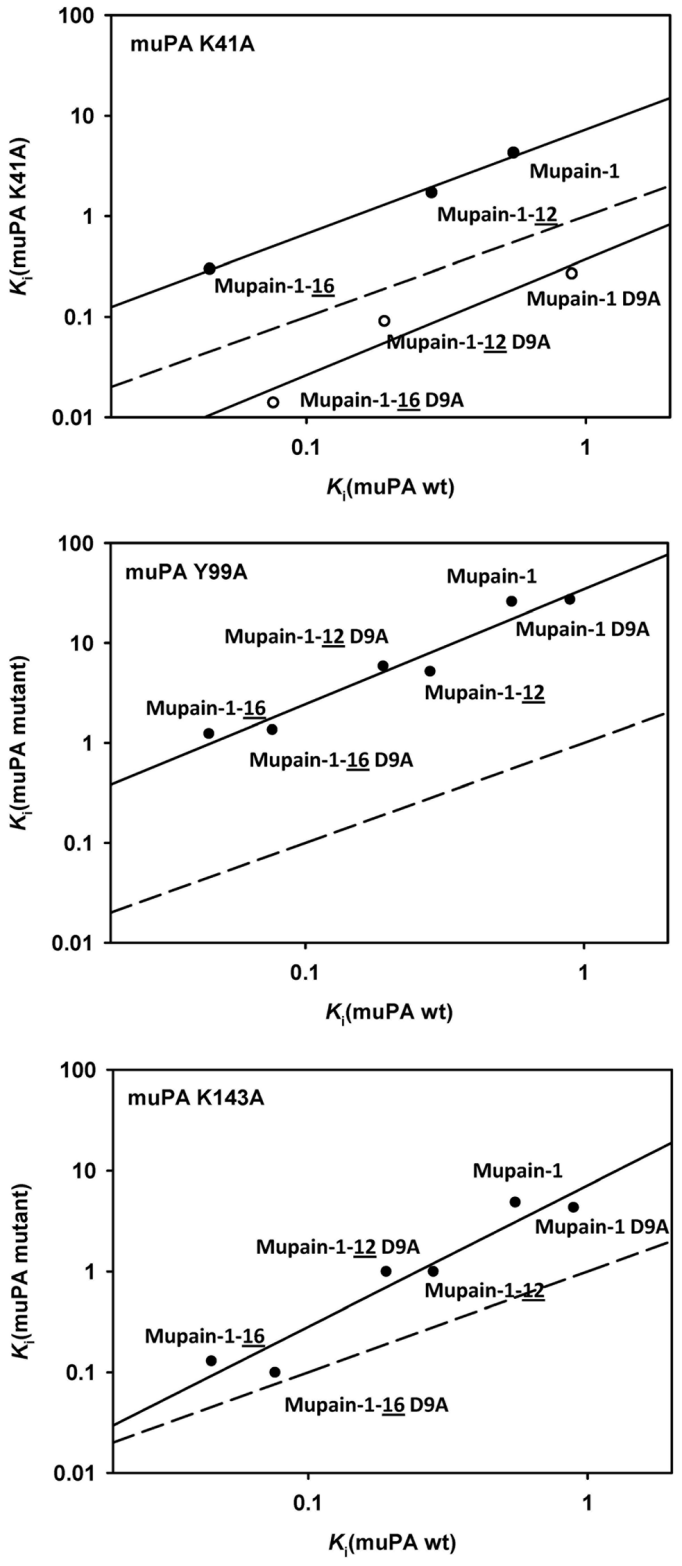
The relationship between the *K*_i_ values for inhibition of muPA exosite mutants and the *K*_i_ value for inhibition of muPA wt. The figure is based on the *K*_i_ values presented in Table 5. The x-axes show the *K*_i_ values for inhibition of muPA wt by the indicated peptides. The y-axes shows the corresponding *K*_i_ values for inhibition of muPA K41A, Y99A, or K143A. The lines resulted from simple linear regression analysis. The slope of the lines are 1.04 (muPA K41A with Asp^9^ peptides); 1.15 (muPA K41A with Ala^9^ peptides); 1.15 (muPA Y99A); 1.40 (muPA K143A). The stippled line shows the line which would have resulted if the *K*_i_ values for exosite mutants and muPA wt had been identical (y = x).

To further visualise the importance of the P1 residues of mupain-1, the *K*_i_ values for inhibition of the muPA exosite mutants K41A, Y99A, and K143A by mupain-1, mupain-1-12, and mupain-1-16 and their D9A variants were plotted logarithmically *versus* the corresponding *K*_i_ values for the wt enzymes (Fig. 5). The above-mentioned difference between the Asp^9^ and the Ala^9^ peptides with respect to inhibition of muPA K41A was obvious in the plot, the data points in an Ala^9^ group falling below the y = x line and the data points for the Aap^9^ group falling above the y = x line. There was no such split with the other muPA mutants. With the K41A and the Y99A mutants, the slopes of the lines defined by the data pairs were relatively close to 1. However, with the K143A mutant, the slopes were around 1.4. This slope corresponds to a 9-fold increase in *K*_i_ for mupain-1 following the K143A mutation and an only 2.9-fold increase in *K*_i_ for mupain-1-16 following the K143A mutation and similar differences when comparing the other peptides. This observation shows that the identity of the amino acid in position 143 influences the effect of a P1 substitution and *vice versa*.

The effects of substituting the P1 residue were also analysed by SPR (Table 2). The increased affinities associated with the unnatural P1 residues were found to be accounted for almost exclusively by lower off-rates.

Taken together, these observations are in agreement with the conclusion that the increased affinity with the two unnatural P1 residues is caused by changes in the binding in the S1 pocket. Interestingly, there seems to be a cross-talk between interactions of the peptide in the S1 pocket and interactions with Lys143.

### Analysis of the importance of the P1 residue by NMR

**Figure 6:**
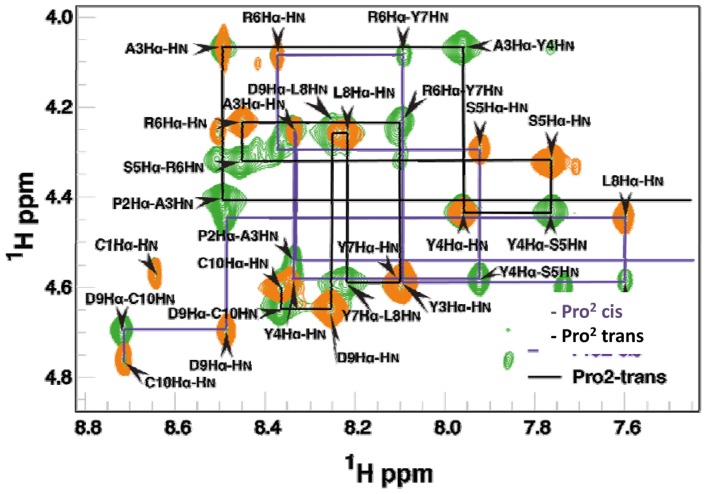
TOCSY (orange) and NOESY (green) of mupain-1-16. Assignments of the two isomeric forms is illustrated by connecting TOCSY H_α_(*i*)-H_N_(*i*) and NOESY H_α_(*i*)-H_N_(*i*+1) cross peaks with lines representing a shared reference commonly referred to as “backbone walks”.

For a further characterization of the importance of the P1 residue, mupain-1 and mupain-1-16 were subjected to ^1^H liquid-state NMR analysis. Analysis of the backbone amide and α-proton region of a TOCSY spectrum revealed a doubling of the expected number of peaks of mupain-1 and mupain-1-16. Following standard procedures for analysis of TOCSY and NOESY spectra [30], the NMR data could be assigned to two markedly different resonance forms, as illustrated by two parallel “backbone walks” connecting sequential residues (Fig. 6). Based on analysis of ^13^C chemical shifts assigned by natural abundance ^13^C-HSQC (difference between C_β_ and C_γ_ chemical shifts) (S8 Table) [31], the two sets of resonances were inferred to be due to *cis*-*trans* isomerization around the Cys^1^-Pro^2^ peptide bond. By integration of isolated peaks in the TOCSY spectrum, the steady state ratio between the two forms was found to be 1∶3 in favor of the *trans* conformation. Since no exchange cross peaks were observed between the two distinct resonance sets corresponding to the two conformers in the time frame of a 200 ms NOESY experiment, the interconversion between the two conformations was estimated to be slower than 1 s^−1^ for both mupain-1 and mupain-1-16. The assigned chemical shifts were close to the random coil values, indicating that the peptides are flexible in solution. Both the proton and carbon chemical shifts for the two isomeric forms were substantially different for most of the residues, suggesting that the average conformations for the *cis* and *trans* isomers are different. It is notable that the spectral differences due to the *cis*-*trans* isomerism propagated throughout the entire peptide chain and not just to the adjacent residues, indicating that the structures are not completely disordered. Furthermore, the chemical shifts for *trans*-mupain-1 and *cis*-mupain-1 are very similar to those of *trans*-mupain-1-16 and cis-mupain-1-16, respectively (as illustrated, *e.g*., by H_α_ in S4 Fig.). This similarity strongly suggests that the structures or average conformations of each of the isomers of each of the two peptides are very similar. Also the similarity in steady state ratio of the *cis*- and *trans*-conformations of the two peptides is in agreement with the notion that the two peptides have the same overall structural and dynamic trends.

Importantly, the presence of the peptides in both a *trans*- and a *cis*-conformation in solution should be contrasted with the fact that only the *trans*-conformation was observed in the crystal structures of the peptide-enzyme complexes.

**Figure 7:**
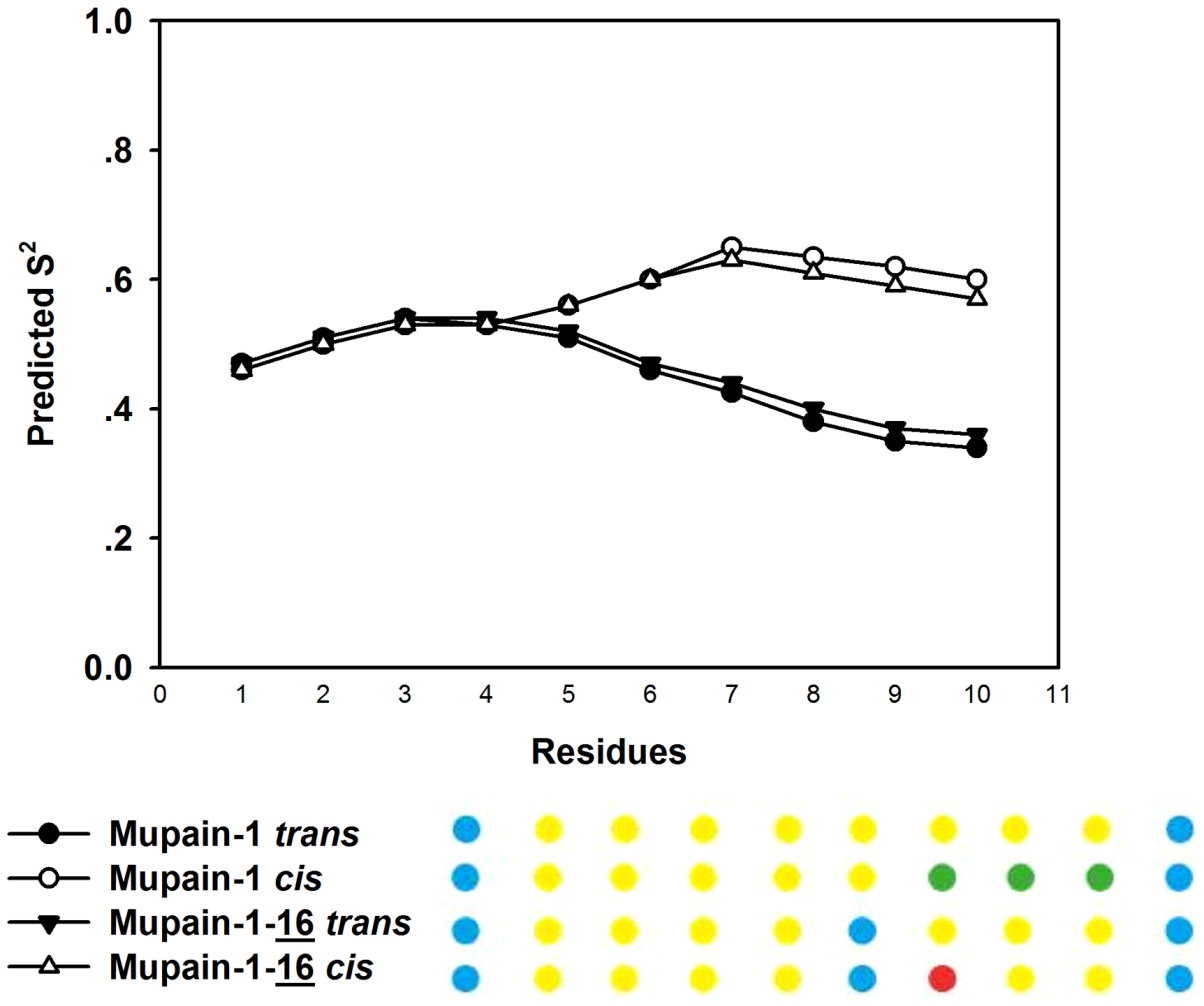
Predicted order parameter S^2^ (top) calculated by TALOS+. The dots (bottom) represent classification of the residue from TALOS+, as based on the mobility of the backbone, the certainty of the angles of the reference triplets and whether the angles fall into allowed regions in the Ramachandran plot [34]. The colour codes represent good (green), dynamic (yellow), ambiguous (red), and no prediction (blue) for the two different peptides in *cis* and *trans* conformations.

The flexibility of the peptides was probed by deriving a predicted order parameter, S^2^, based on the chemical shifts, using random coil index implemented within TALOS+ (Fig. 7). Depending on movements on the picosecond to nanosecond time scale, an S^2^ value of 0 corresponds to a completely disordered peptide and an S^2^ value of 1 to a completely rigid one. The predicted order parameter was found to be relatively low, as also observed from the TALOS+ classification, in which most of the residues fall in the dynamic category.

In summary, the NMR analysis of the peptides in solution did not reveal differences in solution structures between mupain-1 and mupain-1-16.

## Discussion

In this report, we describe studies of the inhibition mechanism and the binding mechanism of derivatives of the serine protease inhibitor mupain-1, which was originally selected from a phage-displayed peptide library for binding to muPA [12]. Besides muPA, we used huPA-H99Y as a model enzyme, because it bound mupain-1 with a reasonable affinity and could, in contrast to muPA, easily be crystallised in complex with the peptides.

Our analyses showed that mupain-1 and derivatives thereof make P1–S1 interactions as well as several exosite interactions with their target proteases. The X-ray crystal structure analysis seemed to yield reliable information about the overall arrangement of the peptide at the enzyme surface and about the inhibitory mechanism of the peptides. There is a good agreement between the X-ray crystal structure analysis and the site-directed mutagenesis analysis as far as the CCPAYS stretch of the peptide is concerned. However, when it comes to the RYLD part of the peptide, it is striking that the effects on the *K*_i_ values of substitution of the P1 residues and of Asp^9^ are not correlated with corresponding changes in the crystal structures. The most likely explanation for this apparent discrepancy is that the peptide is able to sample a number of conformations while bound to the enzyme but that only one is selected during crystallization. It thus seems that site-directed mutagenesis yield information about the details of peptide-enzyme interactions in solution which is not available from X-ray crystal structure analysis. Anyway, the overall arrangement of peptides in the enzyme surface seem to be largely the same in all cases. The *K*_i_ changes observed following site-directed mutagenesis and the differences in *K*_i_ between muPA and huPA-H99Y must reflect relatively small local conformational variations.

The reason for the peptides being inhibitors and not substrates readily became evident from the X-ray crystal structure analysis of the peptide-huPA-H99Y complexes. The distance from the Ser^195^ Oγ to the carbonyl group of the P1 residue of the peptides was found to be too large (>3 Å) to allow the nucleophilic attack associated with catalysis. Moreover, the oxygen atom of the carbonyl group fails to align properly into the oxyanion hole. The conformations of the mupain-1 peptides on the enzyme surface are perpendicular to the previously reported conformation of the peptide upain-1 (CSWRGLENHRMC), a competitive inhibitor of huPA ([20]). In the upain-1 – huPA complex, Glu^7^ of the peptide blocks the oxyanion hole [15], [16], [32]. Thus, the inhibitory mechanism of mupain-1 is different from that of upain-1. An inhibitory mechanism similar to that for upain-1 was observed for two bicyclic peptidic inhibitors of huPA, also with an acidic residue blocking the oxyanion hole [14], [33]. We were unable to crystallise muPA, but the binding mechanism of the peptides to this enzyme was worked out by site-directed mutagenesis. We concluded that the exosite interactions of the CCPAYSR stretch of the peptides are largely the same in their complexes with muPA and huPA-H99Y, respectively, while the exosite contacts made by the YLD stretch of the peptides to each of these two enzymes are likely to differ from those observed by the X-ray crystal structure analysis.

Substituting the P1 Arg of mupain-1 with L-4-guanidinophenylalanine or L-3-(*N*-amidino-4-piperidyl)alanine resulted in a general increase in affinity to muPA and huPA-H99Y as well as several chimeras between muPA and huPA [13]. *A priori*, the explanation for changes in affinity following the P1 substitution could be sought in an energically less favorable solution state or an energically more favorable bound state. We here did a number of observations allowing a distinction between these two possibilities. Firstly, the NMR analysis showed an absence of major differences in the solution structures of mupain-1 and mupain-1-16. The NMR analysis showed that both mupain-1 and mupain-1-16 have a *cis*-*trans* isomerization around the Cys^1^-Pro^2^ peptide bond. In both cases, the bound forms of the peptides had the Cys^1^-Pro^2^ peptide bond in the *trans* conformation, showing that the binding involves a shift in the equilibrium between the conformations towards the *trans*-form. The peptides are quite flexible in solution, so the binding renders the peptides more rigid. These observations argue against a decisive contribution from a difference in solution states of peptides with different P1 residues. Secondly, our mutational analysis showed that a different fit of the side chains of the three different P1 residues into the S1 pockets of the enzymes contributes to the changed affinities. Thirdly, the affinities are also affected by cross-talk between P1-S1 interactions and exosite interactions in mupain-1, involving in particular Lys^143^. It can therefore be concluded that the explanation for the increased affinity of the mupain-1 variants with the unnatural P1 residues is an energetically more favorable bound state rather than an energetically less favorable solution state.

The D9A mutation, unexpectedly, increased the affinity of all tested mupain-1 variants to all tested variants of huPA-H99Y, while there was no effect of the D9A mutation on the affinity to the muPA variants except K41A. In fact, the D9A mutation reduced the difference in affinity of the peptides to huPA-H99Y and muPA wt strongly. It thus seems that the D9A substitution allows mupain-1 to assume more favourable interactions with targets other than the one, against which it was selected. Of particular interest is the fact that the D9A substitution strongly increased the affinity of the peptides to muPA K41A without having any effect on the affinity to the other variants. In fact, the D9A peptides even bound stronger to muPA K41A than to muPA wt. The most ready explanation of this observation is that Lys^41^ of muPA restricts the conformation of the D9A peptide and keeps it from assuming the binding conformation with the lowest energy. Although less striking, the exosite interactions in huPA-H99Y also influenced the effects of the D9A substitution. These observations are in agreement with the notion that the D9A substitution leads to changes in the relative energetic contributions from different exosite interactions. This conclusion is also in agreement with the differential effect of the D9A substitution in muPA and huPA-H99Y, respectively. The D9A mutation makes it easier for the peptide to adopt to the different local environments of muPA and huPA-H99Y in the region around the 37-loop.

The increased affinity following the D9A substitution occurred in spite of an entropy penalty, which was overcome by a more favourable binding enthalpy. The SPR measurements showed that the increased affinity following the D9A substitution was associated with an increased *k*_on_ as well as a decreased *k*_off_. The most ready interpretation of these observations, taken together, is that the Ala^9^ peptides more readily adopt themselves to contacts on the surface of the enzymes than the Asp^9^ peptides. The Ala^9^ peptides may be able to sample a larger conformational space and visit conformations with peptide-enzyme interactions which are inaccessible to the Asp^9^ peptides. Such a larger flexibility of the enzyme-bound Ala^9^ peptides, as compared to the Asp^9^ peptides, is in good agreement with the entropy penalty associated with the D9A substitution, assuming that the entropy increase associated with the increased flexibility is larger in the solution state than in the bound state. Also, the relative B-factor in the huPA H99Y complexes was higher for mupain-1-16 D9A than for mupain-1-16, in agreement with the idea of the D9A peptides being more flexible in the complexs.

Our results show that mupain-1 is an unusual peptidic serine protease inhibitor. Usually, attempts at improving affinity of enzyme inhibitors implicate reduction of the entropic burden associated with binding. This approach is also being pursued with peptidic protease inhibitors [2], [15]. In the present work, another principle was demonstrated. The observation of a strongly increased affinity in spite of an entropy penalty represents a concept going against conventional attempts at improving inhibitor affinity by reducing the entropic burden. Our finding suggests that the D9A versions of mupain-1 may be engineered to target other serine proteases with high affinity. When combined with suitable unnatural amino acids as P1 residues and other measures to assure specificity, the flexibility achieved by the D9A substitution allows it to assume different conformations and make different exosite interactions, resulting in high affinity towards different enzymes. This principle is a new way of engineering inhibitor specificity and affinity.

## Supporting Information

S1 Fig **Alignment of the amino acid sequences of the catalytic domains of muPA and huPA.**
(DOC)

S2 Fig
**Conformation of the bound mupain-1 peptide constrained by two tight 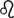-turns and hydrogen bonds.**
(DOC)

S3 Fig **SPR analysis of peptide-enzyme binding.**
(DOC)

S4 Fig **Secondary chemical shifts for the Cα, Cβ, H_N_ and Hα for mupain-1 and mupain-1-16 in** ***cis*** **and** ***trans*** **forms.**
(DOC)

S1 Table **X-ray data collection and model refinement statistics.**
(DOC)

S2 Table ***K*** **_M_ values for S-2444 hydrolysis by muPA and huPA variants.**
(DOC)

S3 Table **Distances between mupain-1 and huPA-H99Y residues in the crystal structure.**
(DOC)

S4 Table **Distances between mupain-1-12 residues and huPA-H99Y residues in the crystal structure.**
(DOC)

S5 Table **Distances between mupain-1-16 residues and huPA-H99Y residues in the crystal structure.**
(DOC)

S6 Table **Distances between mupain-1-16 D9A and huPA-H99Y residues in the crystal structure.**
(DOC)

S7 Table **Comparison of B factors in the huPA-H99Y-mupain-1, huPA-H99Y-mupain-1 -12, huPA-H99Y-mupain-1-16 structures, and huPA-H99Y-mupain-1-16-D9A structures.**
(DOC)

S8 Table **Analysis of ^13^C chemical shifts of mupain-1 and mupain-1-16.**
(DOC)
