# Supplementary material for: A Cyclic Peptidic Serine Protease Inhibitor: Increasing Affinity by Increasing Peptide Flexibility
Source: PLoS One. 2014 Dec 29;9(12):e115872. doi: 10.1371/journal.pone.0115872 (PMC4278837; doi:10.1371/journal.pone.0115872)
Supplement: S1 Fig — Alignment of the amino acid sequences of the catalytic domains of muPA and huPA. (DOC) [file pone.0115872.s001.doc]

**Supporting Figure S1. Alignment of the amino acid sequences of the catalytic domains of muPA and huPA.** Residues implicated in the binding to the peptides by the X-ray crystal structure analysis are highlighted in cyan. The residue numbering is according to the chymotrypsin template. The alignment was performed with Crustal W2. * indicate identity. Similarities are indicated by . or :.

**16 35 41**

**Human CGQKTLRPRFKIIGGEFTTIENQPWFAAIYRRHRGGS-VTYVCGGSLISPCWVISATHCF**

**Murine CGQKALRPRFKIVGGEFTEVENQPWFAAIYQKNKGGSPPSFKCGGSLISPCWVASAAHCF**

******:*******:***** :**********::::*** :: *********** **:*****

**99**

**Human IDYPKKEDYIVYLGRSRLNSNTQGEMKFEVENLILHKDYSADTLAHHNDIALLKIRSKEG**

**Murine IQLPKKENYVVYLGQSKESSYNPGEMKFEVEQLILHEYYREDSLAYHNDIALLKIRTSTG**

***: ****:*:****:*: .* . ********:****: * *:**:**********:. ***

**143**

**Human RCAQPSRTIQTICLPSMYNDPQFGTSCEITGFGKENSTDYLYPEQLKMTVVKLISHRECQ**

**Murine QCAQPSRSIQTICLPPRFTDAPFGSDCEITGFGKESESDYLYPKNLKMSVVKLVSHEQCM**

**:******:*******. :.*. **:.*********..:*****::***:****:**.:***

**192**

**Human QPHYYGSEVTTKMLCAADPQWKTDSCQGDSGGPLVCSLQGRMTLTGIVSWGRGCALKDKP**

**Murine QPHYYGSEINYKMLCAADPEWKTDSCKGDSGGPLICNIEGRPTLSGIVSWGRGCAEKNKP**

**********:. ********:******:*******:*.::** **:********** *:****

**Human GVYTRVSHFLPWIRSHTKEENGLAL**

**Murine GVYTRVSHFLDWIQSHIGEEKGLAF**

************ ** *** ** ***:**
