# Supplementary material for: A Cyclic Peptidic Serine Protease Inhibitor: Increasing Affinity by Increasing Peptide Flexibility
Source: PLoS One. 2014 Dec 29;9(12):e115872. doi: 10.1371/journal.pone.0115872 (PMC4278837; doi:10.1371/journal.pone.0115872)
Supplement: S2 Fig — Conformation of the bound mupain-1 peptide constrained by two tight -turns and hydrogen bonds. (DOC) [file pone.0115872.s002.doc]

**Supporting Figure S2. Conformation of the bound mupain-1 peptide constrained by two tight β-turns and hydrogen bonds.** Selected hydrogen bonds are indicated by dashed lines.

**
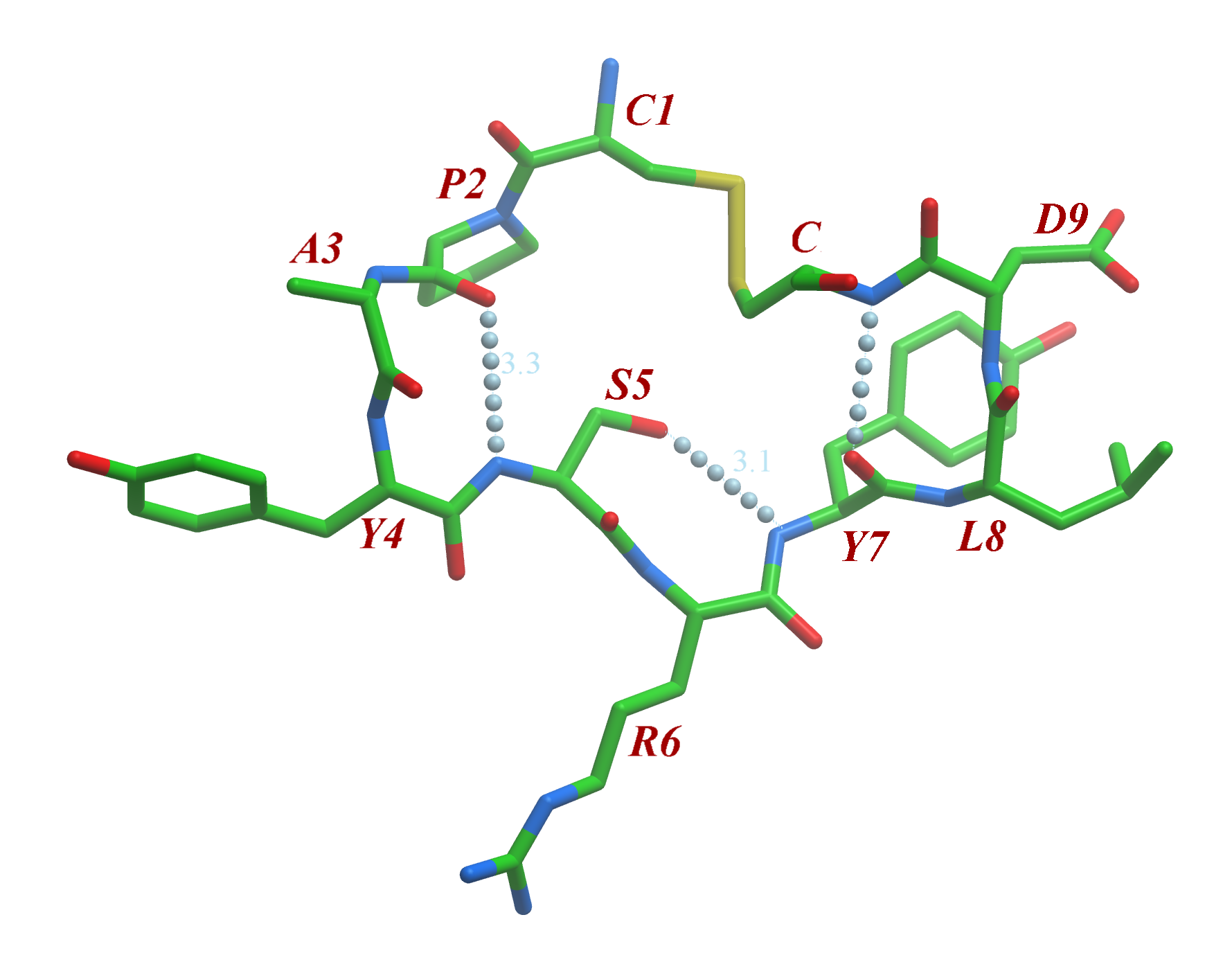
**
