# Supplementary material for: A Cyclic Peptidic Serine Protease Inhibitor: Increasing Affinity by Increasing Peptide Flexibility
Source: PLoS One. 2014 Dec 29;9(12):e115872. doi: 10.1371/journal.pone.0115872 (PMC4278837; doi:10.1371/journal.pone.0115872)
Supplement: S3 Fig — SPR analysis of peptide-enzyme binding. (DOC) [file pone.0115872.s003.doc]

**Supporting Figure S3. SPR analysis of peptide-enzyme binding.** The figure shows representative examples of sensorgrams of analysis of peptide-enzyme binding kinetics. huPA-H99Y was immobilized on a CM5 chip to a level of approximately 500 RU. The peptides were applied to the chip in the following concentration series: Mupain-1, 0.1 – 100 μM; mupain-1-12, 0.025 – 25 μM; mupain-1-16, 0.01 – 10 μM. The experimental sensorgrams are shown in red and the curve fits in black. The fitted curves correspond to *k*_on_ = 0.509 x 10^5^M^-1^s^-1^, *k*_off_ = 80.3 x 10^-2^ s^-1^¸ *K*_D_ = 15.8 μM (mupain-1); *k*_on_ = 1.88 x 10^5^M^-1^s^-1^, *k*_off_ = 24.8 x 10^-2^ s^-1^¸ *K*_D_ = 1.32 μM (mupain-1-12); *k*_on_ = 1.01 x 10^5^M^-1^s^-1^, *k*_off_ = 9.29 x 10^-2^ s^-1^¸ *K*_D_ = 0.920 μM (mupain-1-16). A summary of the results obtained in all experiments performed is given in Table 4 in the main text.

**
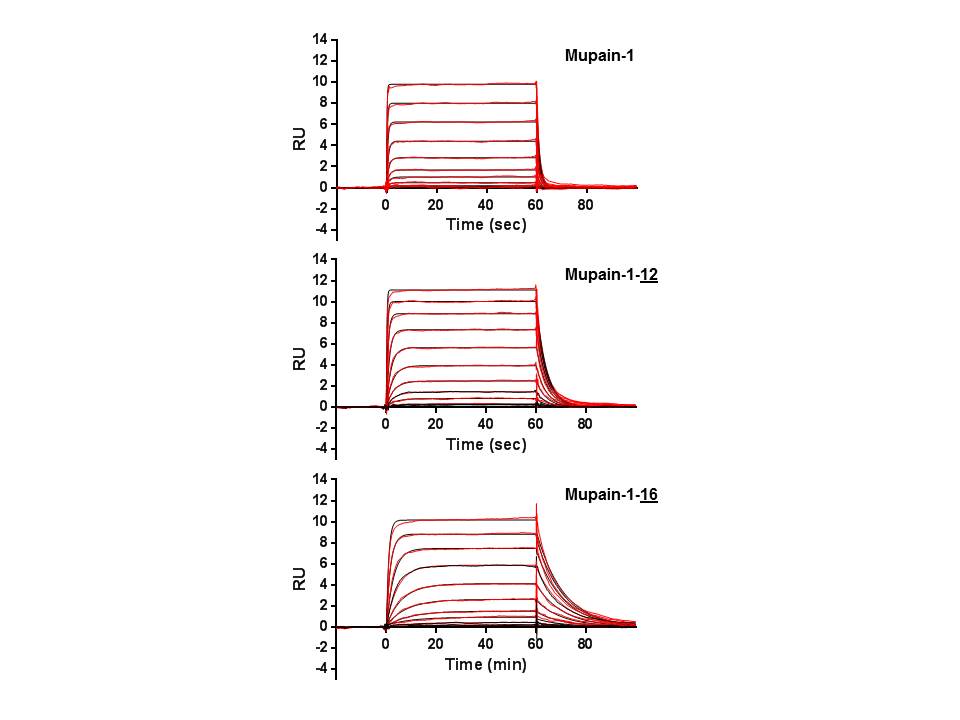
**
