# Supplementary material for: A Cyclic Peptidic Serine Protease Inhibitor: Increasing Affinity by Increasing Peptide Flexibility
Source: PLoS One. 2014 Dec 29;9(12):e115872. doi: 10.1371/journal.pone.0115872 (PMC4278837; doi:10.1371/journal.pone.0115872)

**Supporting Figure S4. Secondary chemical shifts for the Cα, Cβ, H_N_ and Hα for mupain-1 and mupain-1-16 in *cis* and *trans* forms.** The ^1^H and some ^13^C resonances were assigned as described in the main text. Two markedly different assignments for each set of peptide resonances were obtained which was interpreted as *cis*-*trans* isomerization. From the complete assignment of the ^1^H and ^13^C chemical shifts, the secondary chemical shifts for H_α_, H_N_, C_β_ and C_α_ for all the residues were calculated as the assigned chemical shift minus the reference random coil shift. Since the different databases only provide chemical shifts for the 20 standard amino acids, no random coil chemical shift of the non-standard amino acid L-3-(*N*-amidino-4-piperidyl)alanine from mupain-1-16 can be obtained and these values are therefore omitted in the analysis. For the two amino acids next to L-3-(*N*-amidino-4-piperidyl)alanine, Ser^5^ and Tyr^7^, Arg values were used for sequence correction and this substitution should have negligible effects on the backbone chemical shift. Based on the secondary chemical shifts, it is clear, that both the proton and carbon chemical shifts for the two isomeric forms of each peptide are significantly different for most of the residues. The assigned chemical shifts for the side chains also reveal similarities for the same isomeric forms as *e.g.* indicated by the secondary chemical shifts of C_β_.


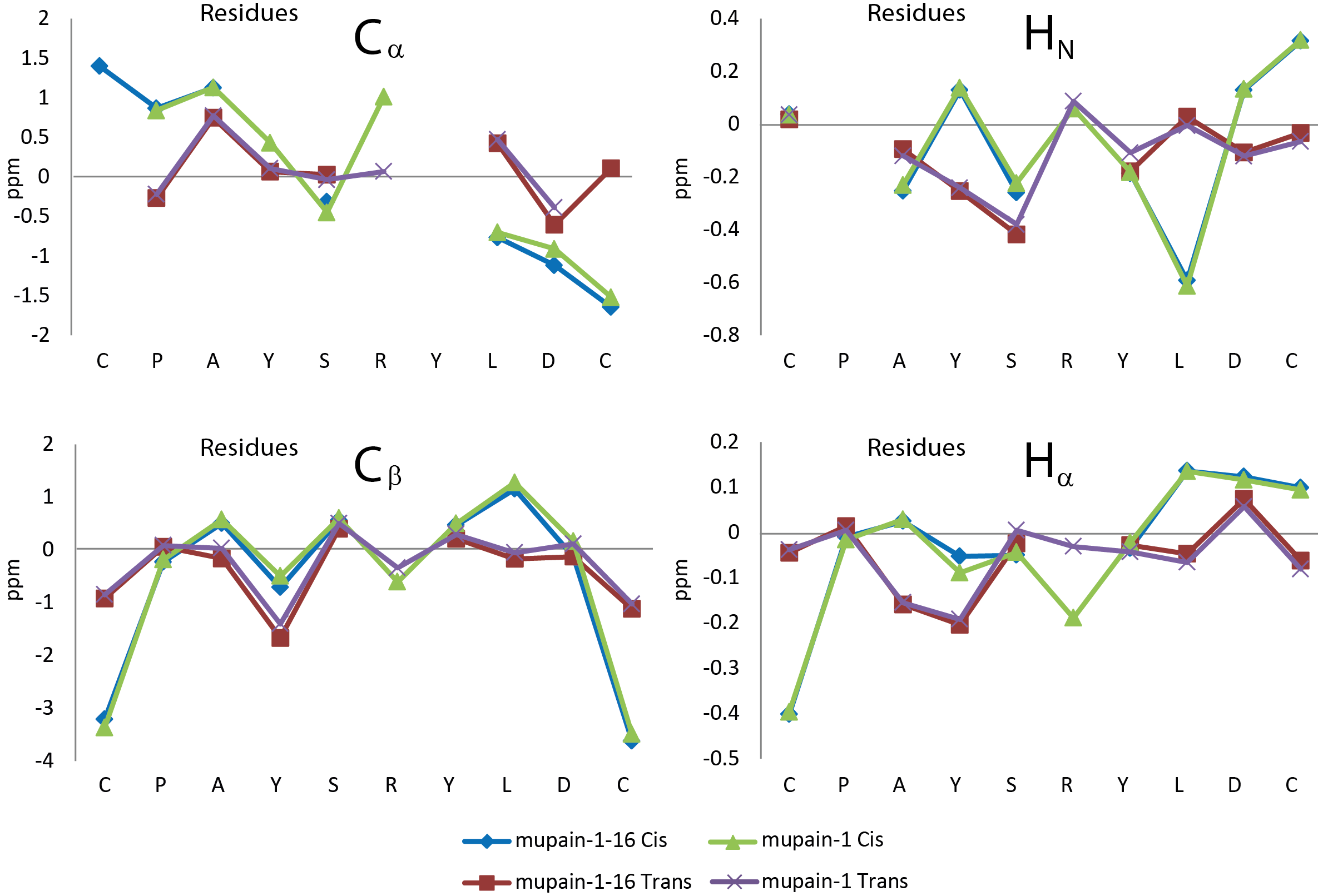

Supplement: S4 Fig — Secondary chemical shifts for the Cα, Cβ, HN and Hα for mupain-1 and mupain-1-16 in cis and trans forms. (DOC) [file pone.0115872.s004.doc]
