# Supplementary material for: A Cyclic Peptidic Serine Protease Inhibitor: Increasing Affinity by Increasing Peptide Flexibility
Source: PLoS One. 2014 Dec 29;9(12):e115872. doi: 10.1371/journal.pone.0115872 (PMC4278837; doi:10.1371/journal.pone.0115872)
Supplement: S1 Table — X-ray data collection and model refinement statistics. (DOC) [file pone.0115872.s005.doc]

| Crystals | huPA-H99Y:mupain-1 at pH 7.4 | huPA-H99Y:mupain-1-12 at pH 7.4 | huPA-H99Y:mupain-1-16 at pH 7.4 | huPA-H99Y:mupain-1-16 D9A at pH 7.4 |
| --- | --- | --- | --- | --- |
| X-ray wavelength (Å) | 1.0 | 1.0 | 1.0 | 1.0 |
| Resolution limits (Å) | 2.28 | 2.10 | 1.80 | 1.9 |
| Space group | R3 | R3 | R3 | R3 |
| Cell parameters (Å) | a=121.11, b=121.11, c=42.55 | a= 121.18, b= 121.18, c= 42.37 | a=121.56, b=121.56, c=42.40 | a=121.30, b=121.30, c=42.93 |
| Temperature of experiments (K) | 100 | 100 | 100 | 100 |
| Completeness (%) | 99.6 | 98.9 | 98.7 | 97.0 |
| Redundancy | 3.6 (3.7)^a^ | 3.4 (3.5)^a^ | 3.8 (3.8)^a^ | 3.4(3.2) |
| Average I/σ | 34.1 (6.0)^a^ | 29.4 (2.8)^a^ | 37.9 (3.0)^a^ | 32.4(2.9) ^a^ |
| R_merge_^b^ | 0.084 (0.517)^a^ | 0.076 (0.570)^a^ | 0.052 (0.478)^a^ | 0.067(0.557) |
| Refinement data |  |  |  |  |
| R-factor | 0.205 | 0.210 | 0.230 | 0.205 |
| R-free | 0.287 | 0.273 | 0.276 | 0.249 |
| Average B-factor (Å^2^) of protein | 43.3 | 57.6 | 39.9 | 37.16 |
| Average B-factor (Å^2^) of peptide | 68.1 | 66.1 | 51.2 | 68.8 |
| r.m.s deviation of bond lengths (Å) | 0.008 | 0.009 | 0.007 | 0.008 |
| r.m.s deviation of angle lengths (°) | 1.230 | 1.285 | 1.230 | 1.111 |
| Ramachandran analysis (%) | 95.2^c^, 4.8^d^, 0^e^ | 96.4^c^, 3.2^d^, 0.4^e^ | 95.6^c^, 4.0^d^, 0.4^e^ | 94.4^c^, 5.2^d^, 0.4^e^ |

**Supporting Table S1. X-ray data collection and model refinement statistics**

^a^ Numbers in parentheses refer to the highest resolution shells.

^b^ Rmerge=Σ|Ii-<I>|/ΣIi, where Ii is the intensity of the ith observation and <I> is the mean intensity of the reflections.

^c^ Percentage of residues in most favored regions.

^d^ Percentage of residues in additional allowed regions.

^e^ Percentage of residues in generously allowed regions.
