# Supplementary material for: A Cyclic Peptidic Serine Protease Inhibitor: Increasing Affinity by Increasing Peptide Flexibility
Source: PLoS One. 2014 Dec 29;9(12):e115872. doi: 10.1371/journal.pone.0115872 (PMC4278837; doi:10.1371/journal.pone.0115872)
Supplement: S2 Table — K M values for S-2444 hydrolysis by muPA and huPA variants. (DOC) [file pone.0115872.s006.doc]

**Supporting Table S2. *K*_M_ values for S-2444 hydrolysis by muPA and huPA variants.** For the following variants of muPA, the *K*_M_ values did not deviate significantly from that of the wt: Q35A; N37A; K37aA; G37cA; S37dA; P37eA; P38A; Q60aA; E146A; Y149A.

| Enzyme | *K*_M_ (mM) |
| --- | --- |
| muPA wt | 2.40 ± 0.20 (3) |
| muPA K41A | 2.50 ± 0.20 (3) |
| muPA Y99A | 3.60 ± 0.30 (3) |
| muPA K143A | 1.00 ± 0.40 (3) |
| muPA S190A | 10.8 ± 1.0 (3) |
| muPA V213T | 0.868 ± 0.350 (3) |
| huPA wt | 0.087 ± 0.0003 (3) |
| huPA-H99Y | 0.610 ± 0.110 (3) |
| huPA-H99Y R35A | 0.517 ± 0.156 (3) |
| huPA-H99Y V41A | 0.742 ± 0.073 (3) |
| huPA-H99Y K143A | 0.500 ± 0.041 (3) |
| huPA-H99Y Q192A | 0.904 ± 0.178 (3) |
