# Supplementary material for: A Cyclic Peptidic Serine Protease Inhibitor: Increasing Affinity by Increasing Peptide Flexibility
Source: PLoS One. 2014 Dec 29;9(12):e115872. doi: 10.1371/journal.pone.0115872 (PMC4278837; doi:10.1371/journal.pone.0115872)
Supplement: S3 Table — Distances between mupain-1 and huPA-H99Y residues in the crystal structure. (DOC) [file pone.0115872.s007.doc]

**Supporting Table S3. Distances between mupain-1 and huPA-H99Y residues in the crystal structure**

| **Mupain-1 residue** | **huPA-H99Y residue** | **Distance, Å** |
| --- | --- | --- |
| Ala^3^ N | Thr^97a^ O | 2.68 |
| Tyr^4^ N | Leu^97b^ O | 3.17 |
| Tyr^4^ OHη | Arg^217^ NHη1 | 3.47 |
| Ser^5^ O | Tyr^99^ OHη | 2.18 |
| Arg^6^ N2 | Gly^219^ O | 2.79 |
| Arg^6^ N2 | Asp^189^ Oδ2 | 2.68 |
| Arg^6^ N1 | Asp^189^ Oδ1 | 3.14 |
| Arg^6^ N1 | Ser^190^ O | 3.33 |
| Arg^6^ N1 | Ser^190^ Oγ | 2.67 |
| Arg^6^ O | Gly^193^ N | 2.84 |
| Tyr^7^ OH | Arg^35^ NHη1 | 3.13 |
| Tyr^7^ OH | Arg^35^ NHη2 | 3.20 |
| Tyr^7^ OH | Cys^58^ O | 2.74 |
| Tyr^7^ O | Gln^192^ Nε2 | 3.03 |
| Leu^8^ O | Gln^192^ Nε2 | 2.67 |
| Asp^9^ Oδ1 | Arg^35^ NHη1 | 2.27 |
| Cys^10^ O | Gln^192^ Nε2 | 3.46 |
