# Supplementary material for: A Cyclic Peptidic Serine Protease Inhibitor: Increasing Affinity by Increasing Peptide Flexibility
Source: PLoS One. 2014 Dec 29;9(12):e115872. doi: 10.1371/journal.pone.0115872 (PMC4278837; doi:10.1371/journal.pone.0115872)
Supplement: S4 Table — Distances between mupain-1-12 residues and huPA-H99Y residues in the crystal structure. (DOC) [file pone.0115872.s008.doc]

**Supporting Table S4. Distances between mupain-1-12 residues and huPA-H99Y residues in the crystal structure**

| **Mupain-1-12 residue** | **huPA-H99Y residue** | **Distance, Å** |
| --- | --- | --- |
| Ala^3^ N | Thr^97A^ O | 2.72 |
| Tyr^4^ O | Gly^216^ N | 2.96 |
| Tyr^4^ N | Leu^97B^ O | 3.28 |
| Ser^5^ Oγ | Tyr^99^ OH | 2.26 |
| [CPAYS[4-guanidino-phenyl-alanine]YLDC]^6^ N1 | Gly^216^ O | 3.52 |
| [CPAYS[4-guanidino-phenyl-alanine]YLDC]^6^ N1 | Gly^219^ O | 2.48 |
| [CPAYS[4-guanidino-phenyl-alanine]YLDC]^6^ N2 | Asp^189^ Oδ1 | 2.88 |
| [CPAYS[4-guanidino-phenyl-alanine]YLDC]^6^ N2 | Ser^190^ Oγ | 2.98 |
| [CPAYS[4-guanidino-phenyl-alanine]YLDC]^6^ N2 | Ser^190^ O | 3.09 |
| [CPAYS[4-guanidino-phenyl-alanine]YLDC]^6^ N3 | Asp^189^ Oδ1 | 3.35 |
| [CPAYS[4-guanidino-phenyl-alanine]YLDC]^6^ N3 | Asp^189^ Oδ2 | 2.63 |
| [CPAYS[4-guanidino-phenyl-alanine]YLDC]^6^ N3 | Gly^219^ O | 3.09 |
| [CPAYS[4-guanidino-phenyl-alanine]YLDC]^6^ O | Gly^193^ N | 3.01 |
| Tyr^7^ OH | Arg^35^ NH1 | 3.37 |
| Tyr^7^ OH | Arg^35^ NH2 | 3.58 |
| Tyr^7^ OH | Cys58 O | 3.01 |
| Tyr^7^ O | Gln^192^ Nε2 | 3.52 |
| Leu^8^ O | Tyr^151^ OH | 3.72 |
| Leu^8^ O | Gln^192^ Nε2 | 2.90 |
| Asp^9^ Oδ2 | Arg^35^ NH1 | 3.26 |
| Cys^10^ O | Gln^192^ Nε2 | 3.27 |
