# Supplementary material for: A Cyclic Peptidic Serine Protease Inhibitor: Increasing Affinity by Increasing Peptide Flexibility
Source: PLoS One. 2014 Dec 29;9(12):e115872. doi: 10.1371/journal.pone.0115872 (PMC4278837; doi:10.1371/journal.pone.0115872)
Supplement: S5 Table — Distances between mupain-1-16 residues and huPA-H99Y residues in the crystal structure. (DOC) [file pone.0115872.s009.doc]

**Supporting Table S5. Distances between mupain-1-16 residues and huPA-H99Y residues in the crystal structure**

| **Mupain-1-16 residue** | **huPA-H99Y residue** | **Distance, Å** |
| --- | --- | --- |
| Ala^3^ N | Thr^97^ O | 2.68 |
| Tyr^4^ N | Leu^97^ O | 2.98 |
| Tyr^4^ OHη | Arg^217^ NHη1 | 2.63 |
| Ser^5^ O | Tyr^99^ OHη | 2.59 |
| [L-3-(*N*-amidino-4-piperidyl)alanine]^6^ N10 | Gly^219^ O | 2.97 |
| [L-3-(*N*-amidino-4-piperidyl)alanine]^6^ N10 | Asp^189^ Oδ2 | 2.73 |
| [L-3-(*N*-amidino-4-piperidyl)alanine]^6^ N9 | Asp^189^ Oδ1 | 2.71 |
| [L-3-(*N*-amidino-4-piperidyl)alanine]^6^ N9 | Ser^190^ O | 3.24 |
| [L-3-(*N*-amidino-4-piperidyl)alanine]^6^ N9 | Ser^189^ Oγ | 2.66 |
| [L-3-(*N*-amidino-4-piperidyl)alanine]^6^ O | Gly^193^ N | 3.10 |
| [L-3-(*N*-amidino-4-piperidyl)alanine]^6^ O | Ser^195^ Oγ | 3.36 |
| Tyr^7^ O | Gln^192^ Nε2 | 3.20 |
| Tyr^7^ OHη | Arg^35^ NHη1 | 2.86 |
| Tyr^7^ OHη | Arg^35^ NHη2 | 2.73 |
| Tyr^7^ OHη | Cys^58^ O | 2.97 |
| Tyr^7^ Oα | Gln^192^ Nε2 | 3.19 |
| Leu^8^ O | Gln^192^ Nε2 | 3.49 |
| Asp^9^ Oδ1 | Arg^36^ NHη1 | 2.53 |
| Cys^10^ Oα | Gln^192^ Nε2 | 2.79 |
