# Supplementary material for: A Cyclic Peptidic Serine Protease Inhibitor: Increasing Affinity by Increasing Peptide Flexibility
Source: PLoS One. 2014 Dec 29;9(12):e115872. doi: 10.1371/journal.pone.0115872 (PMC4278837; doi:10.1371/journal.pone.0115872)
Supplement: S6 Table — Distances between mupain-1-16 D9A and huPA-H99Y residues in the crystal structure. (DOC) [file pone.0115872.s010.doc]

**Supporting Table S6. Distances between mupain-1-16 D9A and huPA-H99Y residues in the crystal structure**

| **Mupain-1-16 D9A residue** | **huPA H99Y residue** | **Distance, Å** |
| --- | --- | --- |
| Ala^3^ N | Thr^97A^ O | 2.53 |
| Tyr^4^ N | Leu^97B^ O | 3.06 |
| Tyr^4^ OHη | Arg^217^ NHη1 | 2.34 |
| Tyr^4^ OHη | Arg^217^ N | 2.50 |
| Tyr^4^ O | Gly^216^ N | 2.90 |
| Tyr^4^ O | Gly^216^ O | 2.88 |
| Ser^5^ O | Tyr^99^ OHη | 2.70 |
| [L-3-(*N*-amidino-4-piperidyl)alanine]^6^ N2 | Gly^219^ O | 2.74 |
| [L-3-(*N*-amidino-4-piperidyl)alanine]^6^ N2 | Ser^190^ O | 3.35 |
| [L-3-(*N*-amidino-4-piperidyl)alanine]^6^ N2 | Asp^189^ Oδ2 | 3.03 |
| [L-3-(*N*-amidino-4-piperidyl)alanine]^6^ N1 | Asp^189^ Oδ1 | 2.96 |
| [L-3-(*N*-amidino-4-piperidyl)alanine]^6^ N1 | Asp^189^ Oδ2 | 3.35 |
| [L-3-(*N*-amidino-4-piperidyl)alanine]^6^ N1 | Ser^190^ O | 3.21 |
| [L-3-(*N*-amidino-4-piperidyl)alanine]^6^ N1 | Ser^190^ Oγ | 2.67 |
| [L-3-(*N*-amidino-4-piperidyl)alanine]^6^ O | Gly^193^ N | 3.07 |
| [L-3-(*N*-amidino-4-piperidyl)alanine]^6^ O | Ser^195^ Oγ | 2.83 |
| Tyr^7^ OH | Arg^35^ NHη1 | 3.17 |
| Tyr^7^ OH | Arg^35^ NHη2 | 3.25 |
| Tyr^7^ OH | Cys^58^ O | 2.91 |
| Tyr^7^ O | Gln^192^ Nε2 | 3.36 |
| Tyr^7^ N | Ser^195^ Oγ | 3.48 |
| Leu^8^ O | Gln^192^ Nε2 | 3.27 |
| Asp^9^ Oδ1 | Arg^35^ NHη1 | 2.27 |
| Cys^10^ O | Gln^192^ Nε2 | 2.90 |
