# Supplementary material for: A Cyclic Peptidic Serine Protease Inhibitor: Increasing Affinity by Increasing Peptide Flexibility
Source: PLoS One. 2014 Dec 29;9(12):e115872. doi: 10.1371/journal.pone.0115872 (PMC4278837; doi:10.1371/journal.pone.0115872)

**Supporting Table S7. Comparison of B factors in the huPA-H99Y-mupain-1, huPA-H99Y-mupain-1 -12, huPA-H99Y-mupain-1-16 structures, and huPA-H99Y-mupain-1-16-D9A structures**

| Compound | Average B factor |
| --- | --- |
| huPA-H99Y-mupain-1 complex | 44.2 |
| mupain-1 in huPA-H99Y-mupain-1 complex | 68.1 |
| huPA-H99Y in huPA-H99Y-mupain-1 complex | 43.3 |
|  |  |
| huPA-H99Y-mupain-1-12 complex | 57.8 |
| mupain-1-12 in huPA-H99Y-mupain-1-12 complex | 66.1 |
| huPA-H99Y in huPA-H99Y-mupain-1-12 complex | 57.6 |
|  |  |
| huPA-H99Y-mupain-1-16 complex | 39.1 |
| mupain-1-16 in huPA-H99Y-mupain-1-16 complex | 48.5 |
| huPA-H99Y in huPA-H99Y-mupain-1-16 complex | 38.7 |
|  |  |
| huPA-H99Y-mupain-1-16-D9A complex | 39.6 |
| mupain-1-16 D9A in huPA-H99Y-mupain-1-16-D9A complex | 55.0 |
| huPA-H99Y in huPA-H99Y-mupain-1-16-D9A complex | 38.9 |
|  |  |
| The ratio of B factor of mupain-1 in huPA-H99Y-mupain-1 complex | 68.1/44.2=1.54 |
| The ratio of B factor of mupain-1-12 in huPA-H99Y-mupain-1-12 complex | 66.1/57.8=1.14 |
| The ratio of B factor of mupain-1-16 in huPA-H99Y-mupain-1-16 complex | 48.5/39.1=1.24 |
| The ratio of B factor of mupain-1-16-D9A in huPA-H99Y-mupain-1-16-D9A complex | 55.5/39.6=1.39 |
| The ratio of B factor of huPA-H99Y in huPA-H99Y-mupain-1 complex | 43.3/44.2=0.98 |
| The ratio of B factor of huPA-H99Y in huPA-H99Y-mupain-12 complex | 57.6/57.8=0.99 |
| The ratio of B factor of huPA-H99Y in huPA-H99Y-mupain-1-16 complex | 38.7/39.1=0.99 |
| The ratio of B factor of huPA-H99Y in huPA-H99Y-mupain-1-16-D9A complex | 38.9/39.6 = 0.98 |

**Supporting Table S8. Analysis of ^13^C chemical shifts of mupain-1 and mupain-1-16.** The chemical shifts and differences between C_β_ and C_γ_ for the Pro residues in mupain-1 and mupain-1-16 for characterization of *cis* and *trans* conformation by the values found by Shubert et al. [2002]. The difference Δ between C_β_ and C_γ_ found by Shubert et al. [2002] was between 0.0 ppm - 4.8 ppm for 100% *trans* and 9.15 ppm - 14.4 ppm for 100% *cis*.

|  | Mupain-1 | | Mupain-1-16 | |
| --- | --- | --- | --- | --- |
|  | *Cis* | *Trans* | *Cis* | *Trans* |
| C_β_(ppm) | 34.39 | 32.06 | 34.35 | 32.04 |
| C_γ_(ppm) | 24.33 | 27.42 | 24.19 | 27.39 |
| Δ(ppm) | 10.06 | 4.64 | 10.16 | 4.65 |

**Supporting Figure S1. Alignment of the amino acid sequences of the catalytic domains of muPA and huPA.** Residues implicated in the binding to the peptides by the X-ray crystal structure analysis are highlighted in cyan. The residue numbering is according to the chymotrypsin template. The alignment was performed with Crustal W2. * indicate identity. Similarities are indicated by . or :.

**16 35 41**

**Human CGQKTLRPRFKIIGGEFTTIENQPWFAAIYRRHRGGS-VTYVCGGSLISPCWVISATHCF**

**Murine CGQKALRPRFKIVGGEFTEVENQPWFAAIYQKNKGGSPPSFKCGGSLISPCWVASAAHCF**

******:*******:***** :**********::::*** :: *********** **:*****

**99**

**Human IDYPKKEDYIVYLGRSRLNSNTQGEMKFEVENLILHKDYSADTLAHHNDIALLKIRSKEG**

**Murine IQLPKKENYVVYLGQSKESSYNPGEMKFEVEQLILHEYYREDSLAYHNDIALLKIRTSTG**

***: ****:*:****:*: .* . ********:****: * *:**:**********:. ***

**143**

**Human RCAQPSRTIQTICLPSMYNDPQFGTSCEITGFGKENSTDYLYPEQLKMTVVKLISHRECQ**

**Murine QCAQPSRSIQTICLPPRFTDAPFGSDCEITGFGKESESDYLYPKNLKMSVVKLVSHEQCM**

**:******:*******. :.*. **:.*********..:*****::***:****:**.:***

**192**

**Human QPHYYGSEVTTKMLCAADPQWKTDSCQGDSGGPLVCSLQGRMTLTGIVSWGRGCALKDKP**

**Murine QPHYYGSEINYKMLCAADPEWKTDSCKGDSGGPLICNIEGRPTLSGIVSWGRGCAEKNKP**

**********:. ********:******:*******:*.::** **:********** *:****

**Human GVYTRVSHFLPWIRSHTKEENGLAL**

**Murine GVYTRVSHFLDWIQSHIGEEKGLAF**

************ ** *** ** ***:**

**Supporting Figure S2. Conformation of the bound mupain-1 peptide constrained by two tight β-turns and hydrogen bonds.** Selected hydrogen bonds are indicated by dashed lines.

**
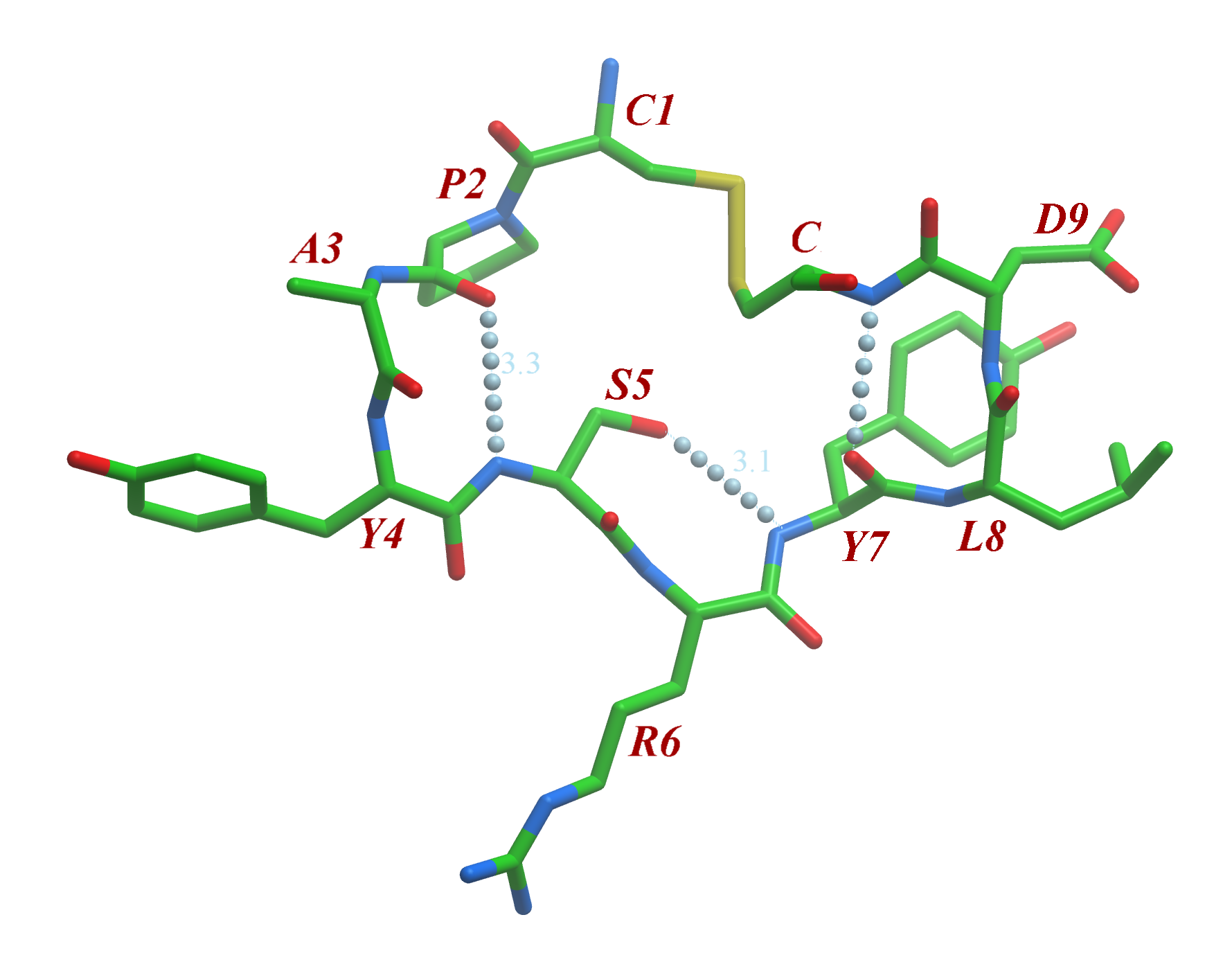
**

**Supporting Figure S3. SPR analysis of peptide-enzyme binding.** The figure shows representative examples of sensorgrams of analysis of peptide-enzyme binding kinetics. huPA-H99Y was immobilized on a CM5 chip to a level of approximately 500 RU. The peptides were applied to the chip in the following concentration series: Mupain-1, 0.1 – 100 μM; mupain-1-12, 0.025 – 25 μM; mupain-1-16, 0.01 – 10 μM. The experimental sensorgrams are shown in red and the curve fits in black. The fitted curves correspond to *k*_on_ = 0.509 x 10^5^M^-1^s^-1^, *k*_off_ = 80.3 x 10^-2^ s^-1^¸ *K*_D_ = 15.8 μM (mupain-1); *k*_on_ = 1.88 x 10^5^M^-1^s^-1^, *k*_off_ = 24.8 x 10^-2^ s^-1^¸ *K*_D_ = 1.32 μM (mupain-1-12); *k*_on_ = 1.01 x 10^5^M^-1^s^-1^, *k*_off_ = 9.29 x 10^-2^ s^-1^¸ *K*_D_ = 0.920 μM (mupain-1-16). A summary of the results obtained in all experiments performed is given in Table 4 in the main text.

**
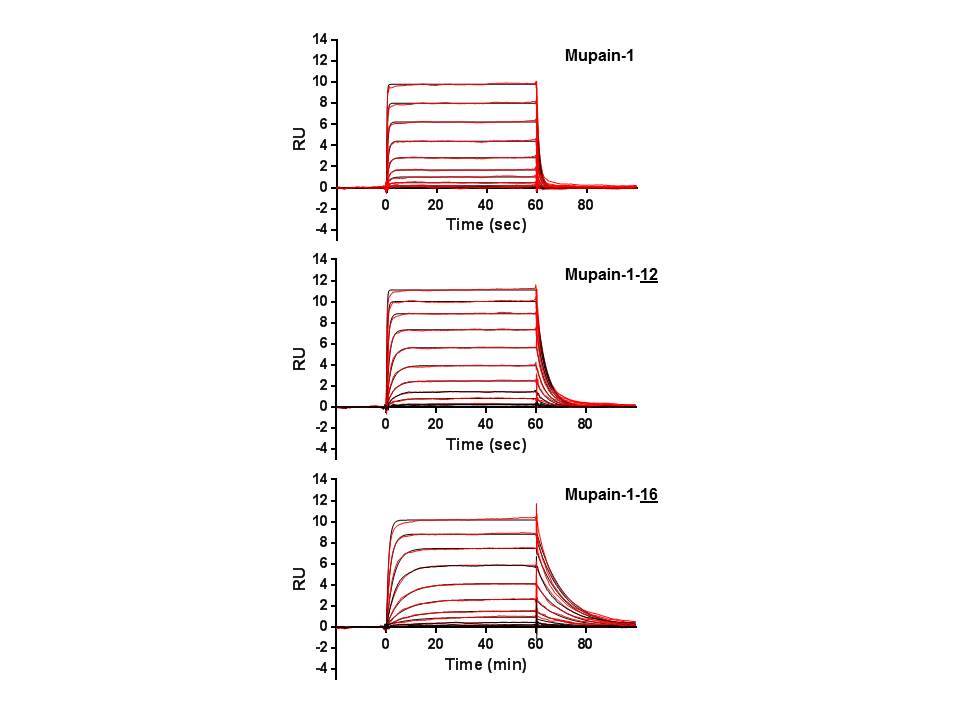
**

**Supporting Figure S4. Secondary chemical shifts for the Cα, Cβ, H_N_ and Hα for mupain-1 and mupain-1-16 in *cis* and *trans* forms.** The ^1^H and some ^13^C resonances were assigned as described in the main text. Two markedly different assignments for each set of peptide resonances were obtained which was interpreted as *cis*-*trans* isomerization. From the complete assignment of the ^1^H and ^13^C chemical shifts, the secondary chemical shifts for H_α_, H_N_, C_β_ and C_α_ for all the residues were calculated as the assigned chemical shift minus the reference random coil shift. Since the different databases only provide chemical shifts for the 20 standard amino acids, no random coil chemical shift of the non-standard amino acid L-3-(*N*-amidino-4-piperidyl)alanine from mupain-1-16 can be obtained and these values are therefore omitted in the analysis. For the two amino acids next to L-3-(*N*-amidino-4-piperidyl)alanine, Ser^5^ and Tyr^7^, Arg values were used for sequence correction and this substitution should have negligible effects on the backbone chemical shift. Based on the secondary chemical shifts, it is clear, that both the proton and carbon chemical shifts for the two isomeric forms of each peptide are significantly different for most of the residues. The assigned chemical shifts for the side chains also reveal similarities for the same isomeric forms as *e.g.* indicated by the secondary chemical shifts of C_β_.


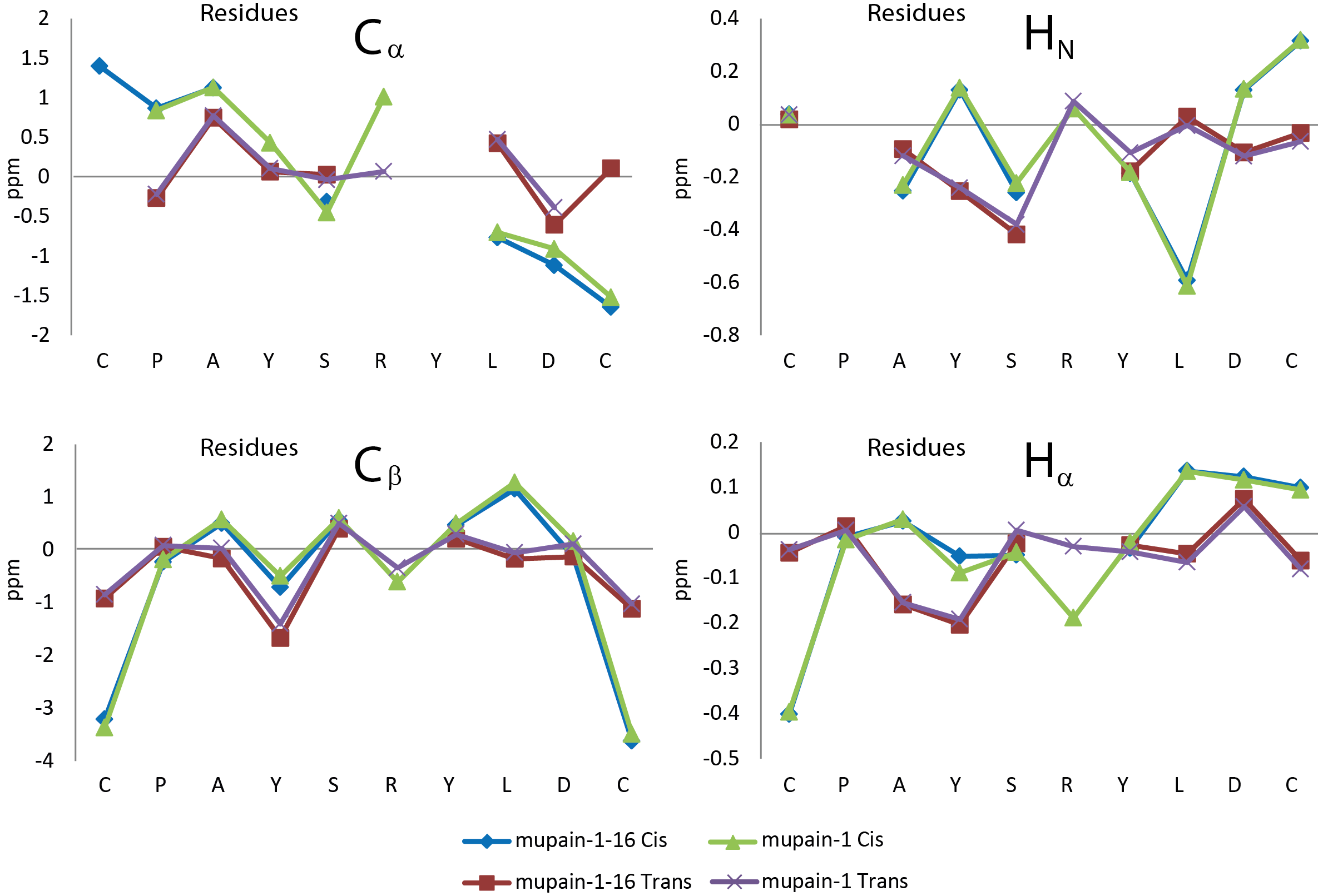

Supplement: S7 Table — Comparison of B factors in the huPA-H99Y-mupain-1, huPA-H99Y-mupain-1 -12, huPA-H99Y-mupain-1-16 structures, and huPA-H99Y-mupain-1-16-D9A structures. (DOC) [file pone.0115872.s011.doc]
