# Supplementary material for: A Cyclic Peptidic Serine Protease Inhibitor: Increasing Affinity by Increasing Peptide Flexibility
Source: PLoS One. 2014 Dec 29;9(12):e115872. doi: 10.1371/journal.pone.0115872 (PMC4278837; doi:10.1371/journal.pone.0115872)
Supplement: S8 Table — Analysis of 13C chemical shifts of mupain-1 and mupain-1-16. (DOC) [file pone.0115872.s012.doc]

**Supporting Table S8. Analysis of ^13^C chemical shifts of mupain-1 and mupain-1-16.** The chemical shifts and differences between C_β_ and C_γ_ for the Pro residues in mupain-1 and mupain-1-16 for characterization of *cis* and *trans* conformation by the values found by Shubert et al. [2002]. The difference Δ between C_β_ and C_γ_ found by Shubert et al. [2002] was between 0.0 ppm - 4.8 ppm for 100% *trans* and 9.15 ppm - 14.4 ppm for 100% *cis*.

|  | Mupain-1 | | Mupain-1-16 | |
| --- | --- | --- | --- | --- |
|  | *Cis* | *Trans* | *Cis* | *Trans* |
| C_β_(ppm) | 34.39 | 32.06 | 34.35 | 32.04 |
| C_γ_(ppm) | 24.33 | 27.42 | 24.19 | 27.39 |
| Δ(ppm) | 10.06 | 4.64 | 10.16 | 4.65 |
